# Mapping microglia and astrocyte activation in vivo using diffusion MRI

**DOI:** 10.1126/sciadv.abq2923

**Published:** 2022-05-27

**Authors:** Raquel Garcia-Hernandez, Antonio Cerdán Cerdá, Alejandro Trouve Carpena, Mark Drakesmith, Kristin Koller, Derek K. Jones, Santiago Canals, Silvia De Santis

**Affiliations:** 1 Instituto de Neurociencias, CSIC/UMH, San Juan de Alicante, Alicante, Spain.; 2 CUBRIC, School of Psychology, Cardiff University, Cardiff, UK.

## Abstract

While glia are increasingly implicated in the pathophysiology of psychiatric and neurodegenerative disorders, available methods for imaging these cells in vivo involve either invasive procedures or positron emission tomography radiotracers, which afford low resolution and specificity. Here, we present a noninvasive diffusion-weighted magnetic resonance imaging (MRI) method to image changes in glia morphology. Using rat models of neuroinflammation, degeneration, and demyelination, we demonstrate that diffusion-weighted MRI carries a fingerprint of microglia and astrocyte activation and that specific signatures from each population can be quantified noninvasively. The method is sensitive to changes in glia morphology and proliferation, providing a quantitative account of neuroinflammation, regardless of the existence of a concomitant neuronal loss or demyelinating injury. We prove the translational value of the approach showing significant associations between MRI and histological microglia markers in humans. This framework holds the potential to transform basic and clinical research by clarifying the role of inflammation in health and disease.

## Teaser

Diffusion-weighted MRI carries the fingerprint of glia activation and can image brain inflammation in vivo and noninvasively.

## INTRODUCTION

Brain diseases with a degenerative component such as Alzheimer’s, Parkinson’s, multiple sclerosis, and dementia are a pressing problem for developed societies with aging populations (*1*–*3*). Accumulating evidence suggests chronic neuroinflammation, the sustained activation of microglia and astrocytes, to strongly influence neurodegeneration and contribute to its progression. A major question is whether inhibition of the inflammatory response has the ability to reverse or slow down its symptoms (*4*). In addition, abnormal immune activation during puberty and adolescence has been associated with increased vulnerability to brain disorders later in life (*5*), making the characterization of the inflammatory profile along the life span a hot topic. Therapies targeting glial cells are currently being proposed as disease-altering treatments to improve the outcome of neurological disorders, with extremely promising results (*4*, *6*, *7*). Furthermore, for many brain diseases, neuroinflammation is emerging as a cause, rather than a consequence, of the pathogenesis (*8*); thus, characterizing the tissue inflammatory state could provide valuable early disease biomarkers. In this context, desired properties of such biomarkers would be the capacity to detect both changes in morphology and proliferation/depletion (both hallmarks of glia activation), and to discriminate inflammation with and without neurodegeneration. In addition, the set of biomarkers should show a response specific to glia, which can be teased apart from the response to other tissue insults relevant in some neurodegenerative diseases, e.g., demyelination.

While imaging techniques are widely adopted to monitor neurological conditions, noninvasive approaches able to specifically characterize brain inflammation in vivo are lacking. The current gold standard is positron emission tomography (PET)–based targeting of the 18-kDa translocator protein. While difficult to generalize due to different binding genotypes across individuals (*9*), PET is associated with ionizing radiation exposure, which limits its use in vulnerable populations and longitudinal studies, and also has low spatial resolution, making it unsuitable to image small structures. In addition, while PET’s main advantage relates to molecular specificity of tracer binding, inflammation-specific radiotracers express across multiple cell types (microglia, astrocytes, and endothelium). Last, a significant tracer uptake in the periphery makes it hard to separate central from peripheral inflammation (*10*). Diffusion-weighted magnetic resonance imaging (dw-MRI), on the other hand, has the unique ability to image brain microstructure in vivo, noinvasively, and with high resolution by capturing the random motion of water molecules in brain parenchyma (*11*).

While the dw-MRI signal is potentially sensitive to all extracellular and intracellular spaces that restrict water displacement in the tissue, current formulations are mostly designed for white matter and axons. A few recent studies, establishing the groundwork for this work, showed that conventional MRI signal can be sensitive to various alterations in microglia (*12*–*14*), but none so far showed specificity to microglia and astrocyte activation, or inflammation in the presence of neurodegeneration. Achieving specificity is of key importance as neurodegenerative diseases manifest through different mechanisms, involving specific cell populations, all playing potentially different roles in disease causation and progression. By combining advanced dw-MRI sequences with mathematical models based on neurobiological knowledge of brain parenchyma morphology, the diffusion characteristics within specific tissue compartments, and even cell types, could be measured (*15*).

With this idea in mind, we developed an innovative strategy to image microglia and astrocyte activation in gray matter using dw-MRI, by building a microstructural multicompartment tissue model informed by knowledge of microglia and astrocyte morphology. To validate the model, we first used an established rat paradigm of inflammation based on intracerebral lipopolysaccharide (LPS) administration (*16*). In this paradigm, neuronal viability and morphology are preserved, while inducing microglial activation within a few hours, and a delayed astrocytic response that is detectable only 24 hours after injection (*17*). Therefore, glial responses can be transiently dissociated from neuronal degeneration, and the signature of reactive microglia investigated independently of any astrogliosis. Then, to isolate the imaging fingerprint of astrocyte activation, we repeated the LPS experiment but pretreating the animals with the CSF1R inhibitor PLX5622 (Plexxikon Inc.), which is known to temporally deplete around 90% of the microglia (*18*). Then, we used an established paradigm of neuronal damage, based on ibotenic acid administration (*19*), to test whether the model was able to disentangle neuroinflammatory signatures with and without a concomitant neurodegeneration. This is pivotal for demonstrating the utility of the framework as a biomarker discovery platform for the inflammatory status in neurodegenerative diseases, where both glia activation and neuronal damage are key players. Last, we used an established paradigm of demyelination, based on focal administration of lysolecithin (*20*), to demonstrate that the developed biomarkers are not confounded by tissue alterations frequently found in brain disorders and with a strong impact on water diffusivity.

We demonstrate that the dw-MRI signal carries the fingerprint of microglial and astrocyte activation, with signatures specific to each glia population, reflecting the morphological changes as validated postmortem by quantitative immunohistochemistry. We demonstrate that the framework is both sensitive and specific to inflammation with and without neurodegeneration so that the two conditions can be teased apart. In addition, we demonstrate that the biomarker set can discriminate between inflammation and demyelination, supporting the possibility to interrogate and characterize brain parenchyma with compartment-specific biomarkers. We demonstrate the translational value of the approach in a cohort of healthy humans at high resolution, in which we performed a reproducibility analysis. Statistically significant association with known microglia density patterns in the human brain supports the utility of the method to generate reliable glia biomarkers. A framework able to characterize relevant aspects of tissue microstructure during inflammation, in vivo and noninvasively, is expected to have a tremendous impact on our understanding of the pathophysiology of many brain conditions and transform current diagnostic practice and treatment monitor strategies.

## RESULTS

### Microstructural model of diffusion and immunological challenge in rats

We built a model of gray matter diffusivity, as detailed in Materials and Methods. Briefly, the model accounts for water diffusion in the microglial compartment corresponding to small cell somas, modeled as small spheres, with thin cellular processes, modeled as sticks, growing radially with a dispersion captured by a dispersion parameter (according to a Watson distribution of orientations), and an astrocytic compartment consisting of large globular cells, modeled as large spheres (*21*). Note that while glial fibrillary acidic protein (GFAP) stains the cytoskeleton of the cell, astrocytes have a globular shape (*22*). Both compartments are embedded in an extracellular space compartment, composed of a tensor-like subcompartment (hindered water in contact with structures and cells) and a free water compartment of water undergoing unrestricted diffusion. All MRI markers analyzed (stick fraction, stick dispersion, small and big sphere radius, and tissue fraction) are defined in Materials and Methods. Changes in the tissue fraction (the reciprocal of the free water signal) are interpreted as a surrogate measure of tissue loss, i.e., of degeneration (*23*). We then tested the mathematical model with three experimental paradigms. In the first one, neuroinflammation without neurodegenerations is induced by an injection of LPS; in the second, neuroinflammation with degeneration is induced by an injection of ibotenic acid; in the third one, we induced demyelination using injections of lysolecithin, which causes a transient, rapidly resolving inflammation and a long-lasting demyelination (at least 3 weeks) without remyelination. All injections were performed in one hemisphere targeting the dentate gyrus of the hippocampus, with the contralateral side as within-subjects control (vehicle injected; see Materials and Methods for details).

### Microglia activation characterized using Iba-1 staining and MRI

**Fig. 1.**
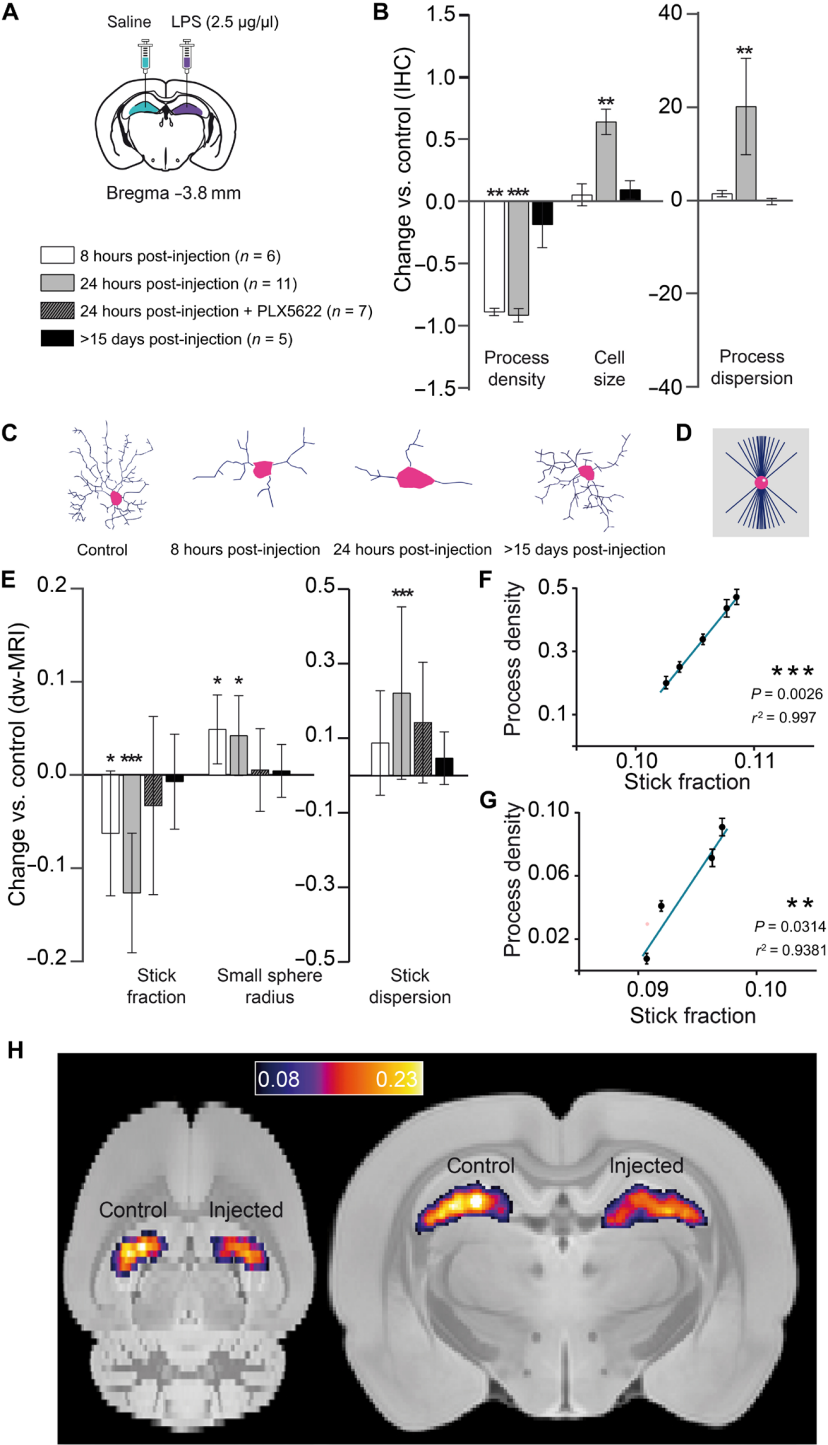
Histological characterization of microglia reaction and its associated MRI signature. (**A**) Experimental scheme showing bilateral stereotaxic injection of LPS (left hemisphere)/saline (right hemisphere) and the composition of the four groups: Six animals were scanned 8 hours after injection, 11 animals were scanned 24 hours after injection, 7 animals were treated with PLX5622 for 7 days before the injection and then scanned 24 hours after injection, and 5 animals were scanned 15 days or more after injection. (**B**) Normalized change (*P*_injected_ − *P*_control_)/*P*_control_ in process density, cell size, and process dispersion parameter for the injected versus control hippocampus, measured in Iba-1^+^–stained microglia for the different groups. Asterisks represent significant paired difference between injected and control (**P < 0.01 and ***P < 0.001). Error bars represent SD. IHC, immunohistochemistry. (**C**) Morphology reconstruction of representative microglia at the different times. (**D**) Geometrical model used for microglia. (**E**) Normalized change (*P*_injected_ − *P*_control_)/*P*_control_ between MRI parameter calculated in the injected versus control hemisphere for the microglia compartment. Asterisks represent significant paired difference between injected and control (*P < 0.05 and ***P < 0.001). (**F** and **G**) Correlations between stick fraction from MRI and process density from Iba-1 at 8 (F) and 24 hours after injection (G). (**H**) Mean stick fraction maps at 24 hours after injection, normalized to a rat brain template and averaged over all rats.

Morphological analysis of Iba-1^+^ cells by histology in the tissue at different time points after LPS injection demonstrated a fast microglial reaction with retraction of cellular processes at 8 hours, which progressed at 24 hours with an additional increase in the microglial cell body size and an increase in the process dispersion parameter (indicating less dispersion), as shown in Fig. 1 (B and C). No changes in cell density are found (fig. S6). The distinct and time-dependent changes in microglial cell morphology were tightly mirrored by the imaging parameters specifically related to the microglia compartment, i.e., sticks (cellular processes) and the small spheres (cell soma), as shown in Fig. 1E. Accordingly, the stick fraction was significantly reduced at 8 hours in the injected versus control hippocampus and further reduced at 24 hours. The stick dispersion parameter was increased at 24 hours, and the radius of the small sphere component was significantly increased at 8 and 24 hours. These LPS-induced changes disappeared when the animals were depleted of microglia by pretreatment with PLX5622 (fig. S7), demonstrating the specificity of these MRI signatures to microglia. Moreover, when looking at interindividual variability, a strong association was found between MRI-derived microglial marker change and their histological counterparts at all measured time points (Fig. 1, F and G). Last, 2 weeks after the injection, when complete recovery was expected, all parameters measured from both histology and MRI converged, showing no statistically significant difference between injected and control hemispheres. Overall, these results demonstrated the possibility to recover a microglia-specific signal from dw-MRI with capacity to unmask a microglial reaction in vivo (Fig. 1H). Whole grey matter average map of the stick fraction is reported in fig. S8.

### Astrocyte activation characterized using GFAP staining and MRI

**Fig. 2.**
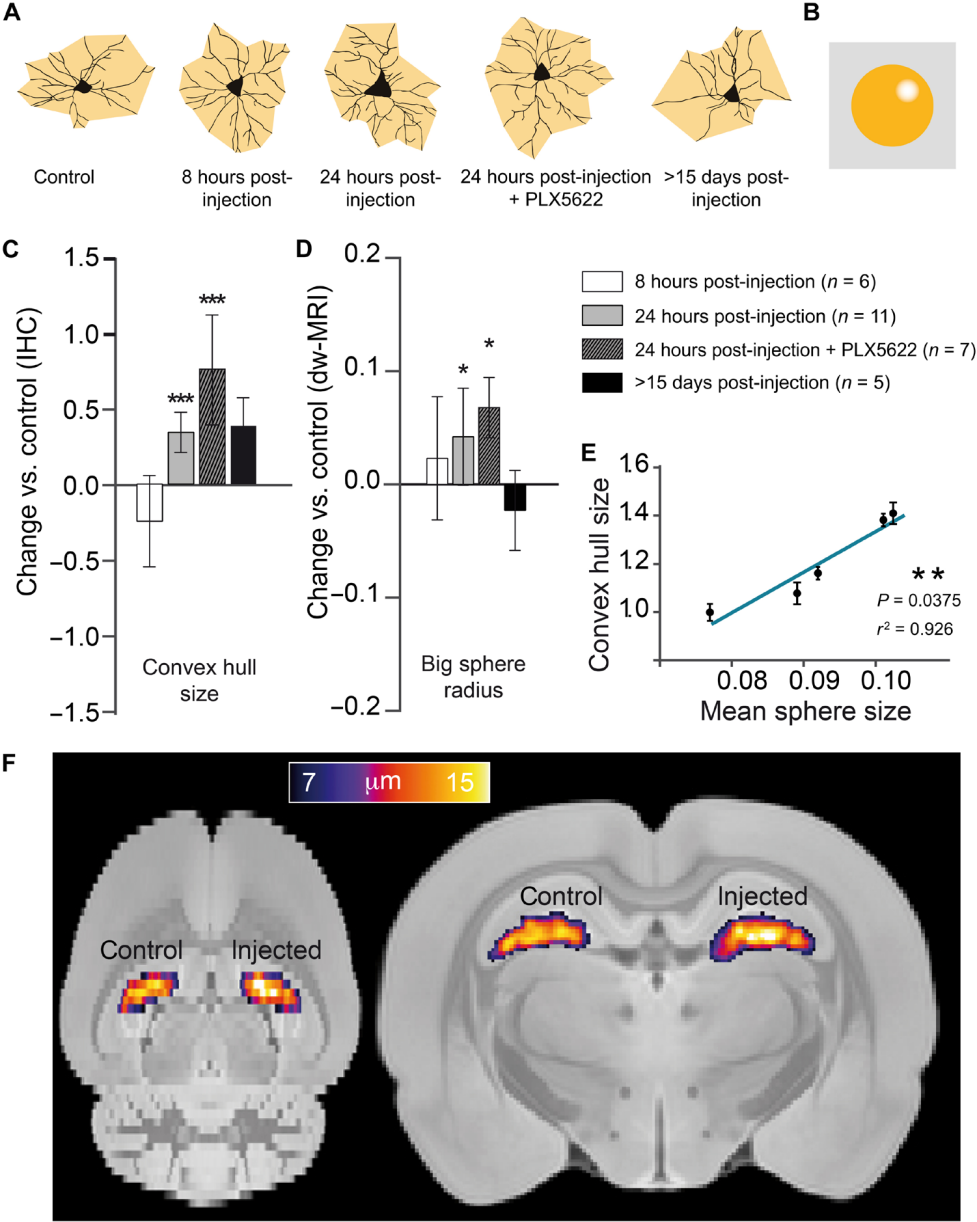
Histological characterization of astrocyte reaction and its associated MRI signature. (**A**) Morphology reconstruction of representative astrocytes at the different times in black and two-dimensional (2D) convex hull in orange. (**B**) Geometrical model used for astrocytes. (**C**) Normalized change (*P*_injected_ − *P*_control_)/*P*_control_ in convex hull mean radius for the injected versus control hippocampus, measured in GFAP^+^-stained astrocytes for the different groups. Asterisks represent significant paired difference between injected and control (***P < 0.001). Error bars represent SD. (**D**) Normalized change (*P*_injected_ − *P*_control_)/*P*_control_ between MRI-derived large sphere radius calculated in the injected versus control hemisphere for the astrocyte compartment (shown in the inset). Asterisks represent significant paired difference between injected and control (*P < 0.05). (**E**) Correlation between mean sphere radius from MRI and convex hull mean radius from GFAP. (**F**) Large sphere radius maps at 24 hours after injection, normalized to a rat brain template and averaged over all rats.

We next performed a comparable analysis with astrocytes (labeled as GFAP^+^ cells), taking advantage of the distinct time course of their response to LPS injection. This cell population, unlike microglia, showed no significant alterations in either density or morphology at 8 hours after LPS injection, as shown in Fig. 2 (A and C). However, at 24 hours, astrocytes grow in volume as measured by the mean radius of the convex hull (Fig. 2C; see Materials and Methods for details). The associated MRI compartment for astrocytes, i.e., the large spheres, followed the same pattern of changes across conditions (Fig. 2D). Volume of GFAP^+^ cells and the mean radius of the large spheres measured by dw-MRI grew in parallel at 24 hours after LPS injection, were insensitive to microglia depletion with PLX5622, and recovered toward baseline levels at 15 days after injection. Accordingly, their interindividual variability showed a strong correlation (Fig. 2E). Therefore, the results obtained for the astrocytic component also demonstrate the possibility of recovering an astrocyte-specific signal from dw-MRI and the capacity to map astrocytic reactions in vivo (Fig. 2F).

### Concomitant microglia activation and neuronal death characterized using NeuN staining and MRI

**Fig. 3.**
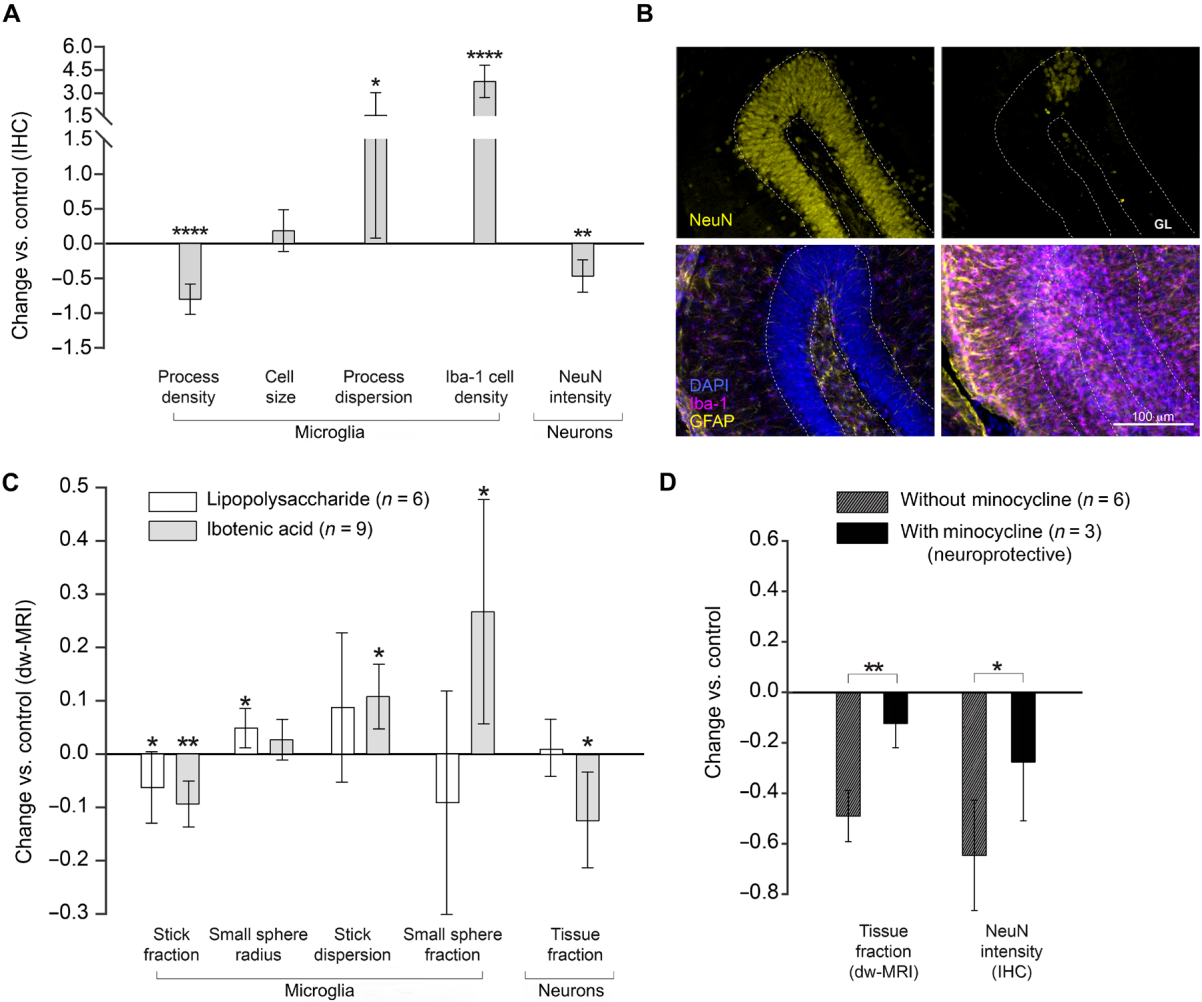
Characterization of inflammation in the presence of neuronal death. (**A**) Normalized change (*P*_injected_ − *P*_control_)/*P*_control_ in histological measures for the injected versus control hippocampus. Asterisks represent significant paired difference between injected and control (*P < 0.05, **P < 0.01, and ****P < 0.0001). (**B**) NeuN and GFAP–Iba-1 staining of a representative animal (left, control; right, injected). GL, granular layer. (**C**) Normalized change (*P*_injected_ − *P*_control_)/*P*_control_ in MRI parameter calculated in the ibotenic-injected versus control hemisphere for microglia and neuron compartments (light gray). For comparison, the same parameters obtained in group 2 of the LPS-injected animals are reported in white. Asterisks represent significant paired difference between injected and control (*P < 0.05 and **P < 0.01). (**D**) Normalized change (*P*_injected_ – *P*_control_)/*P*_control_ for MRI and histological markers of neuronal death calculated separately in the untreated animals and in those treated with minocycline. Asterisks represent significant unpaired difference between the two groups (*P < 0.05 and **P < 0.01).

To challenge the capability of the developed model to distinguish between pure inflammation and inflammation with concomitant neurodegeneration, a cohort of animals was injected as before, but with ibotenic acid, using the contralateral (right) hemisphere as control (saline injected). Histological staining demonstrated that ibotenic acid at the chosen concentration induced a microglial reaction characterized by retraction of the cellular processes with increased dispersion parameter and a significant increase in cell density (Fig. 3, A and B). No alterations in astrocytes were observed, as shown in the Supplementary Materials (fig. S9). Neuronal staining with NeuN unveiled a large decrease in staining intensity in the injected hemisphere, demonstrating the severe neuronal loss induced by ibotenic acid (*19*). These histological findings were tightly mirrored by the MRI parameters (Fig. 3C), with a significant decrease of stick fraction, increase in the stick dispersion parameter, and, notably, increase in the small sphere fraction. In Fig. 3C, the MRI parameters measured in the LPS cohort at 8 hours after injection, where we detected microglia activation but no neuronal damage (Fig. 1), are shown here in white to facilitate the comparison of hallmarks of a glia reaction with and without neuronal damage. Furthermore, a distinct signature for microglia proliferation, captured by the small sphere fraction, differentiating LPS from ibotenic lesions, could be extracted from the dw-MRI data (Fig. 3C).

The tissue fraction component was significantly decreased in the injected hemisphere, compared to control, and only for the ibotenic acid injection not for LPS, suggesting an association with neuronal degeneration. To test this hypothesis, we pretreated the animals with minocycline, an anti-inflammatory drug (*24*), and repeated the ibotenic acid injections as before. NeuN staining demonstrated the protective effect of minocycline on ibotenic-induced neuronal death, and the MRI parameter tissue fraction captured this effect, showing a significant reduction of the ibotenic-induced decrease in this parameter. These results demonstrated the utility of the tissue fraction component to monitor neuronal degeneration.

### Specificity of the model in the presence of demyelination

**Fig. 4.**
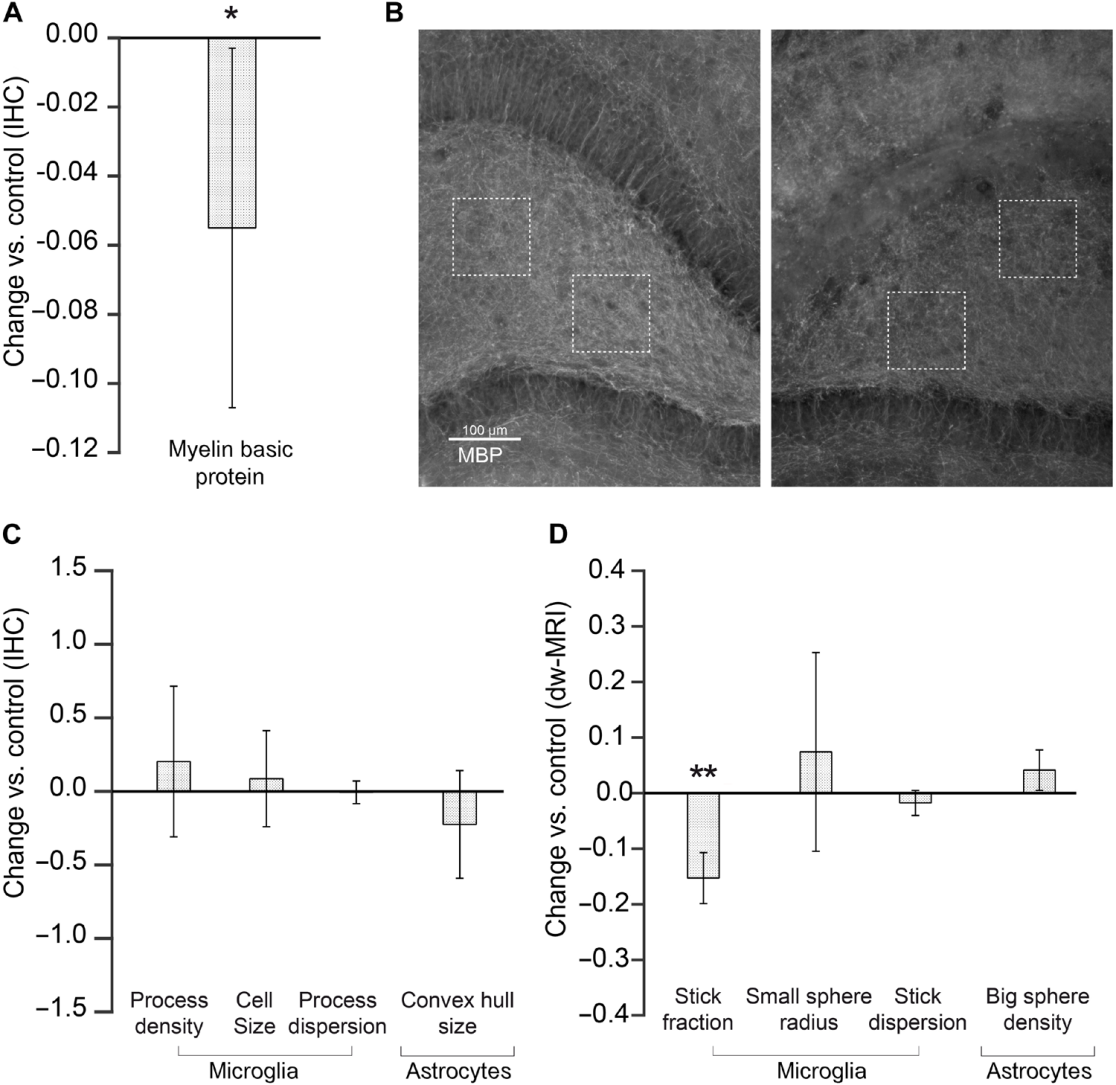
Specificity of glia biomarkers in demyelinated tissue. (**A**) Normalized change (*P*_injected_ − *P*_control_)/*P*_control_ in histological Myelin basic protein measures for the injected versus control hippocampus. (**B**) MBP staining of a representative animal (left, control; right, injected). (**C**) Normalized change (*P*_injected_ − *P*_control_)/*P*_control_ in histological staining calculated in the lysolecithin-injected versus control hemisphere for microglia and astrocyte compartments. (**D**) Same as (C) but for MRI parameters.

A reduction in the myelin content can occur in some brain disorders, which could challenge the specificity of the model. To investigate this possibility, we demyelinated the hippocampus in one hemisphere with an injection of lysolecithin and waited until the induced inflammation was resolved. Demyelination was confirmed as a decrease in the myelin basic protein content in the injected hemisphere (Fig. 4A) and resolved inflammation with glia staining showing no morphological changes (Fig. 4C). In agreement with our model (see below), the only parameter from the MRI biomarker that showed some sensitivity to demyelination was the microglia-associated stick fraction (Fig. 4D). However, demyelination and microglia activation were efficiently discriminated by considering the other microglia compartment parameters, namely, the stick dispersion parameter and the small sphere radius. This finding was further supported by the simulation in fig. S10.

### Comparison with conventional MRI techniques

To highlight the importance of the developed framework, it is important to show that conventional MRI is sensitive to morphological changes due to inflammation, but cannot disentangle the different populations involved across different conditions, as shown in fig. S4. Glia activation, neuronal loss, and demyelination all caused an increase in mean diffusivity, but different conditions could not be differentiated. A clear reduction of T_1_/T_2_ is observed in all three conditions, while histology demonstrates no myelin change (fig. S3). T_2_* does not have enough sensitivity to reflect glia morphological changes at any stages, but a significant reduction is observed in the presence of neuronal loss.

### Translation to human

**Fig. 5.**
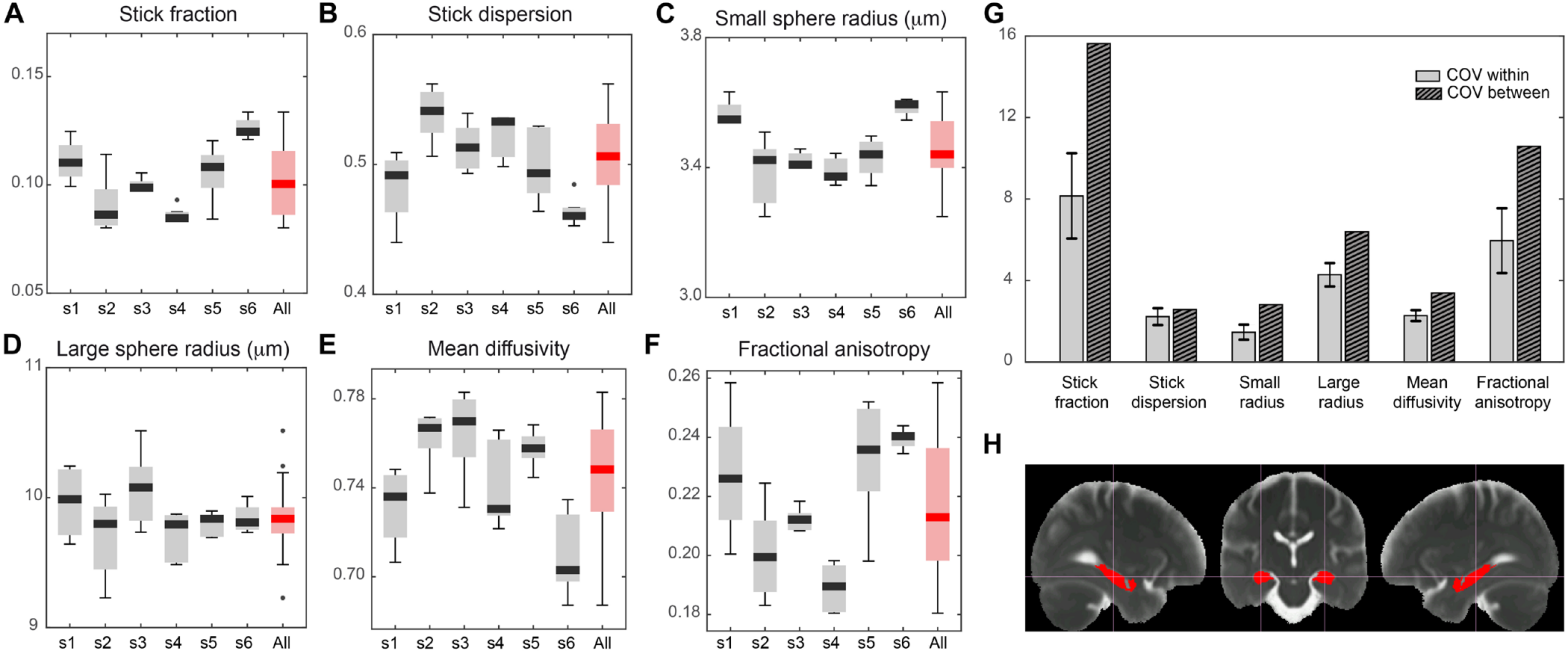
Feasibility of the framework translation to human and MR parameter reproducibility analysis. (**A**) Boxplot of stick fraction as measured separately in the hippocampus of six subjects scanned five times (s1 to s6) and pooling all subjects together (red). The same is shown for the stick dispersion parameter (**B**), small sphere radius (**C**), large sphere radius (**D**), mean diffusivity (**E**), and fractional anisotropy (**F**). (**G**) Average coefficient of variation calculated within subject (light gray) and between subjects (striped). (**H**) Region of interest (ROI) in the hippocampus used for the reproducibility analysis, defined according to (*56*).

**Fig. 6.**
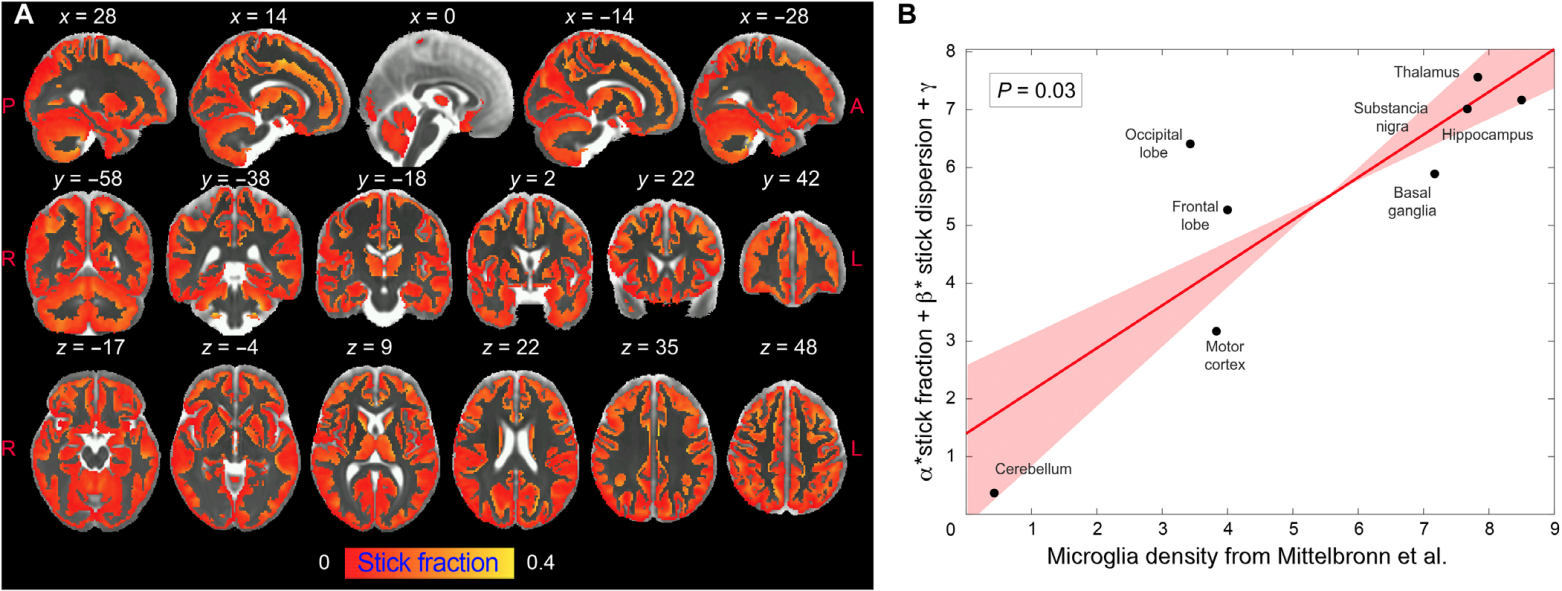
Correlation between the stick fraction and microglia density in human brain. (**A**) Stick fraction according to the MCM normalized to the brain template defined in (*56*), masked for gray matter tissue, and averaged across subjects. (**B**) Multiple linear regression using stick fraction and dispersion to explain microglia density measured using histological staining of postmortem human tissue in eight gray matter regions (hippocampus, cerebellum, substantia nigra, basal ganglia, thalamus, motor, frontal, and occipital cortices) as reported in (*26*). Regression confidence intervals are calculated using bootstrap.

As a proof of concept for the translational validity of these results and to evaluate the reproducibility of the proposed imaging framework, we adapted the MRI protocol to a human 3T Connectom scanner (*25*) and acquired data from a healthy cohort at high resolution (2 mm isotropic). As shown in Fig. 5, the multicompartment model (MCM) applied to these data returned values for the within-subjects coefficient of variations (CoV) in the range of 1.5 to 8% and between-subjects CoV in the range of 2.6 to 15%, which are in the range of conventional MRI measures routinely used in the clinics with diagnostic value. Last, we took advantage of the known heterogeneous distribution of microglial cell densities across brain regions in humans to test the ability of our framework to quantify microglial cell populations in vivo. We found that the patterns of microglia cell density measured postmortem in humans across different gray matter regions (*26*) can be explained by two microglia-related MRI parameters, the stick fraction and the stick dispersion parameter. A multiple linear regression showed a significant correlation between histological and MRI measures (*P* = 0.03; Fig. 6).

## DISCUSSION

Diffusion MRI signal has great potential to reveal the inflammatory component in numerous brain conditions (*27*), and several efforts have been made to provide microstructural models able to capture features belonging to distinct tissue subcompartments, for example, by including dendrite dispersion (*28*, *29*) or a compartment for the soma of neurons (*30*). While specificity to glia can be achieved, to some extent, by looking at diffusion of brain metabolites using diffusion-weighted magnetic resonance spectroscopy (*31*), to date, no imaging framework is available to specifically look at the cellular signature of glia activation. Here, we propose and validate a strategy to image microglia and astrocyte activation in gray matter using diffusion MRI and demonstrate its translational validity to humans. By taking advantage of the different activation windows of glia in an LPS-driven immunological challenge in rats, and by using pharmacological tools to deplete microglia in the brain, we were able to dissect the MRI signatures of specific glial responses. We identified three MRI parameters, namely, the stick fraction, the stick dispersion, and the small sphere size, which, combined, provide sensitivity to, and only to, microglia activation. Similarly, the large sphere size is sensitive to astrocyte activation. In addition, using injections of ibotenic acid, we demonstrate that the framework can distinguish between glia activation and proliferation, independently of an underlying neurodegenerative process. Glia proliferation is an important aspect of the inflammatory reaction and a major component in the evolution of chronic neurodegeneration (*32*). Modulating ibotenic acid–induced neurodegeneration with anti-inflammatory pretreatments, we further unveil an MRI parameter with capacity to monitor neuronal loss. The possibility of teasing apart the contribution of inflammation and neuronal loss has direct implications for understanding the contribution of the brain innate immune response to disease progression, where both components are key players in the pathophysiology and can be targeted by disease-modifying treatments.

Last, we tested the robustness of the developed biomarker set for inflammation in a condition of demyelination. We found that, even in a condition of severe demyelination only comparable to multiple sclerosis, microglia-associated MRI parameters such as the stick dispersion parameter and the small sphere size, and the astrocyte-associated big sphere size, were not confounded. This result, which allows differentiating demyelination and inflammation, is expected not only from basic geometric reasoning but also from our in silico simulation (Supplementary Materials). The MRI stick fraction was, however, decreased by demyelination. This result was expected as myelin is invisible to diffusion-weighted sequences, but its loss causes an increase in the extracellular water fraction. This reweighting of the volume fractions is well characterized in white matter, where the restricted fraction is used as an index sensitive to demyelination in multiple sclerosis (*33*, *34*), and can be easily corrected by including myelin-specific sequences (*35*). This result provides important previously undisclosed information for the interpretation of the volume fractions in dw-MRI.

Our results are supported by quantitative cell morphology analysis. The validation of MRI results is challenging due to several factors, including the need to co-register regions of interest (ROIs) with very different sizes and properties and the need for tissue fixation in histological preparations (*36*). To overcome these limitations, here, we relied on measuring changes rather than absolute magnitude of quantities. We demonstrate that there is a very high correlation between the changes found in injected versus control regions, as measured using MRI and histology in the rat hippocampus, suggesting that our imaging measures truly capture the hallmark of glia activation with high sensitivity. Future work is needed to confirm this finding in the whole brain, possibly exploring the combined use of different antibodies (e.g., NDRG2 and S100-beta for astrocytes) (*37*, *38*) and of other compounds/conditions eliciting inflammation. Furthermore, while the present methodology can detect morphological changes that indicate a status of LPS- or ibotenic-induced activation, future work is needed to dissect the functional status of the different glia populations (*39*).

The proposed MRI methodology was adapted to a human MRI scanner, and healthy subjects were recruited to perform a reproducibility study, demonstrating that the glia biomarkers are highly reproducible between different MRI sessions and in line with CoVs calculated for conventional MRI parameters routinely used in clinical settings (*40*). In vivo variability of MRI-derived microglia biomarkers can explain known patterns of cell density measured postmortem in humans across several regions of the brain parenchyma. While further work is needed to test the model in the presence of, e.g., a neurodegenerative condition, the results establish the value of the developed MRI framework to quantify glial reactions noninvasively in humans.

Our results have implications in the interpretation of several imaging studies published so far. On one hand, we propose that since diffusion MRI signal is sensitive to glia activation, the biological substrate of some of the alterations reported in numerous brain conditions could be driven by changes in glia morphology, rather than the conventional interpretation as “neural damage or degeneration.” For example, multiple sclerosis causes not only demyelination and neuronal damage but also inflammation (*2*), which, according to our results, is likely contributing to the observed differences in MRI parameters between control and patients. On the other hand, our results obtained with traditional MRI, like the mean diffusivity, show that conventional parameters are sensitive to morphological changes due to inflammation, but cannot disentangle the different populations or compartments involved (microglia, astrocytes, neurons, and myelin) (*41*). This implies that the biological substrate of the observed changes is invisible to conventional MRI.

This study has some limitations. The MCM does not explicitly differentiate between microglia processes and neuronal dendrites. However, basic geometrical reasoning supports the idea that axon and dendrite reorganization would affect the stick dispersion parameter in a different way than microglia process retraction and would thus be easily distinguished from a microglia activation. We support this idea using in silico simulations, included as the Supplementary Materials (fig. S10), which show that while microglia process retraction leads to significant increase in the dispersion parameter *k*, dendrite loss leads to a significant decrease. Further experimental data will be required to validate this result. Nevertheless, the MRI results obtained in the animal cohort, which received injection of ibotenic acid, demonstrated that the proposed framework is already capable of distinguishing between glia activation with and without neuronal loss, the fundamental pathological feature of neurodegenerative diseases.

Water exchange between compartments has not been taken into account in the model under a slow exchange assumption, supported by recent literature (*42*). While future work is needed to confirm this assumption, or possibly refine the model through the use of more advanced acquisition and analysis protocols (*43*, *44*), the strong association found between histology and MRI indicates that the contribution of exchange between compartments has to be little in our experimental paradigms.

The injection procedure used to deliver the toxins or saline in the brain is expected to induce a low-grade inflammation just by mechanical trauma. While all precautions have been taken to minimize such damage (inserting slowly the syringe and let it rest before injecting, analyzing a large volume around the injection), we cannot exclude that the area adjacent to the injection scan in both control and experimental hemispheres presents a light inflammatory phenotype. However, all the results are reported as changes in experimental versus control hemispheres, rather than absolute values; this highlights the net effect of the toxin (toxin + injection versus injection alone).

Last, the voxel size for human imaging, while much smaller than most dw-MRI studies (2 mm versus 2.5 to 3 mm), still allows for certain partial volume effects with adjacent structures, which we, however, mitigated by eroding by one voxel the ROIs, by parcellating the brains into gray and white matter, and by retaining only the voxels with negligible white matter content. In general, given the caveats of implementing dw-MRI experiments with varying diffusion time in humans (fundamental to estimate compartment sizes) in regular clinical scanners, future studies addressing translation of the experimental protocol for different hardware configurations are needed.

To conclude, we proposed here a new generation of noninvasive glia-centric biomarkers, which are expected to transform the study of many diseases associated with a glial response: those where inflammation is as a known or possible cause, as well as those in which the glial reaction can serve as a powerful early diagnostic and/or prognostic marker.

## MATERIALS AND METHODS

### Animal preparation

All animal experiments were approved by the Institutional Animal Care and Use Committee of the Instituto de Neurociencias de Alicante, Alicante, Spain, and comply with the Spanish (law 32/2007) and European regulations (EU directive 86/609, EU decree 2001-486, and EU recommendation 2007/526/EC). Rats were housed in groups (*4*, *5*), with 12-hour/12-hour light/dark cycle, lights on at 8:00, at room temperature (23 ± 2°C) and free access to food and water. Glia activation was achieved by intracranial injection in the dorsal hippocampus (coordinates: bregma, −3.8 mm; superior-inferior, 3.0 mm; 2 mm from midline in the left hemisphere) of 2 μl of saline and LPS at a concentration of 2.5 μg/μl. The opposite hemisphere was injected with the same amount of saline. In a cohort of animals, microglia depletion was achieved by administering the CSF1R inhibitor PLX5622 (Plexxikon Inc.) in two ways: as a dietary supplement in standard chow at 1200 ppm (Research Diets) and with an intraperitoneal injection of 50 mg/kg in vehicle with a dose volume of 10 ml/kg once a day for 7 days. Another cohort of rats received the same chow without enrichment and was injected intraperitoneally once a day with the same doses of vehicle. Less than 24 hours after the last injection, all the rats were injected LPS according to the procedure described above. Neuronal death was achieved by injecting 1 μl of saline and ibotenic acid at a concentration of 2.5 μg/μl in the dorsal hippocampus (same coordinates). The opposite hemisphere was injected with the same amount of saline. A subgroup of animals underwent minocycline treatment [45 mg/kg dissolved in phosphate-buffered saline (PBS) at a concentration of 13.5 mg/ml; Sigma-Aldrich, Madrid, Spain] according to (*19*). Minocycline was administered intraperitoneally 12 hours before the surgery, 30 min before the surgery, and once a day for 3 days at 24-hour intervals. Demyelination was achieved by injecting 1 μl of saline and lysolecithin at a concentration of 1% in the dorsal hippocampus (same coordinates). The opposite hemisphere was injected with the same amount of saline.

After different post-injection delays, the rats were scanned in the MRI scanner and immediately perfused for ex vivo MRI immunohistological analysis of Iba-1^+^ and GFAP^+^. A total of 43 rats were used, with weights in the range of 250 to 300 g, divided in six groups. Sample size was chosen on the basis of power calculation performed using, as expected effect size, the mean diffusivity changes reported in rat GM in a previous study looking at inflammation (*45*).

Group 1 (*n* = 6) received the LPS injection and was scanned and perfused after 8 hours. Group 2a (*n* = 7) received the LPS injection and was scanned and perfused after 24 hours. Group 2b (*n* = 4) was treated with control chow and injected with vehicle for 7 days, then received the LPS injection, and was scanned and perfused after 24 hours. Group 3 (*n* = 7) was treated with PLX5622 for 7 days, then received the LPS injection, and was scanned and perfused after 24 hours. Group 4 (*n* = 5) received the LPS injection and was scanned and perfused after a minimum of 15 days or more if the ventricular enlargement was not reabsorbed. No statistically significant differences were detected between groups 2a and 2b (control for PLX5622 chow), so they were merged into a single group for the rest of the analysis. Groups 5a and 5b (*n* = 9) received an ibotenic acid injection and were scanned and perfused at 14 days after surgery. Group 5b (*n* = 6) was treated with minocycline for 5 days. Last, group 6 (*n* = 5) received lysolecithin injection and was scanned and perfused within 2 to 3 weeks. Experimental design is reported in fig. S1.

### MRI experiment

#### Rats

MRI experiments on rats were performed on a 7-T scanner (Bruker, BioSpect 70/30, Ettlingen, Germany) using a receive-only phase array coil with integrated combiner and preamplifier in combination with an actively detuned transmit-only resonator. Dw-MRI data were acquired using an Echo Planar Imaging diffusion sequence, with 30 uniform distributed gradient directions, *b* = 2000 and 4000 s/mm^2^, diffusion times 15, 25, 40, and 60 ms with four images without diffusion weight (*b* = 0, called B0), repetition time (TR) = 7000 ms, and echo time (TE) = 25 ms. Fourteen horizontal slices were set up centered in the hippocampus with field of view (FOV) = 25 mm × 25 mm, matrix size = 110 × 110, in-plane resolution = 0.225 mm × 0.225 mm, and slice thickness = 0.6 mm. In addition, three relaxometry sequences were acquired with the same geometry of the dw-MRI scan: a gradient echo sequence with TR = 1500 ms, 30 TE equally spaced between 3.3 and 83.4 ms, and 3 averages; a T_1_-weighted sequence with TR = 300 ms, TE = 12.6 ms, and 2 averages; and a T_1_-weighted sequence with TR = 3000 ms, TE = 7.7 ms, and 4 averages. Last, a high-resolution anatomical scan with full brain coverage was acquired with TR = 8000 ms, TE = 14 ms, 4 averages, FOV = 25 mm × 25 mm, matrix size = 200 × 200, in-plane resolution = 0.125 mm × 0.125 mm, and 56 slices of thickness = 0.5 mm. Total scan time including animal positioning was around 2 hours.

#### Humans

Six healthy subjects were scanned five times in a 3T Siemens Connectom scanner, for a total of 30 acquisitions. The study was approved by the local Institutional Review Board. Dw-MRI data were acquired using an Echo Planar Imaging diffusion sequence with the following parameters: TE = 80 ms; TR = 3.9 s; diffusion times 17.3, 30, 42, and 55 ms; *b* values of 2000 and 4000 s/mm^2^, each with 30 and 60 uniformly orientated gradient directions, respectively; and six B0 images per diffusion time, yielding a total 384 images. Additional parameters used were as follows: flip angle, 90°; slice thickness, 2 mm; in-plane voxel size, 2 mm; FOV, 220 mm × 220 mm; matrix size, 110 × 110. Total scan time was around 40 min per subject.

### MRI analysis and statistics

Rat MRI data were processed as follows. Preliminary data were used to verify the reach of a 2-μl injection, confirming that the liquid filled the whole dentate gyrus. So, ROIs for the analysis were manually drawn in the dentate gyrus of the dorsal hippocampus; the injection trace was used to locate the central slice, and ROIs were drawn from two slices before the injection, and up to two slices after, for a total length of 3 mm covered. Visual check assured that the injection scar was limited to the central slice only and excluded from the analysis heavily damaged brains and/or brains with enlarged ventricles (one animal was excluded from group 1, one from group 2, one from group 5b, and one from group 6).

Raw dw-MRI data were nonlinearly registered to the T_2_-weighted scan to correct for Echo-planar Imaging distortions, corrected for motion and eddy current distortions using affine registration, and then fed to a custom routine written in MATLAB (R2018a, the MathWorks), which fits the signal to an MCM of diffusion. The MCM is inspired by the AxCaliber model for white matter (*15*) but is adapted to gray matter morphology. The model comprises one compartment of water undergoing restricted diffusion in cylindric geometry (representing water trapped into cell ramifications and axons) with negligible radius; a main orientation and a Watson dispersion term (*29*); two spherically restricted compartments (*46*); one extracellular space matrix, aligned with the main cylinder orientation and modeled as a tensor; and one free water compartment, modeled according to (*23*, *29*). The main cylinder orientation is necessary to pick up the small degree of anisotropy present in gray matter due to axons (*47*). Hence, the signal is expressed by the following expression:

$$S=f_{\text{IC}}\times S_{\text{IC}}(k)+f_{\text{SS}}\times S_{\text{SS}}(R_{\text{SS}})+f_{\text{LS}}\times S_{\text{LS}}(R_{\text{LS}})+f_{\text{EC}}\times S_{\text{EC}}+(1‐f_{T})\times S_{\text{FW}}$$

where *f*_IC_ is the fraction of water undergoing restricted diffusion in cylinders, called stick fraction throughout the paper; *S*_IC_ is the signal in Watson-dispersed cylinders expressed according to equation 2 of (*26*); *k* is the Watson dispersion parameter, called stick dispersion parameter throughout the paper; *f*_SS_ and *f*_LS_ are the fractions of water undergoing restricted diffusion in the two spherical compartments; *S*_SS_ and *S*_LS_ are the signals of water undergoing restricted diffusion in spheres expressed according to equation 18 of (*46*), which depend on the radii of the two spherical compartments *R*_SS_ and *R*_LS_, respectively; *f*_EC_ is the fraction of water hindered in the extracellular space; *S*_IC_ is the signal in the extracellular space modeled as a tensor with radial symmetry, whose main orientation is linked to the main orientation of the cylindrical compartment; 1-*f*_T_ is the fraction of free water (defined as one minus the tissue fraction *f*_T_ to help the discussion); and *S*_FW_ is the free water signal, defined as in (*23*). The fitting parameters are *f*_IC_, *f*_SS_ and *f*_LS_, *k*, *R*_SS_ and *R*_LS_, the extracellular tensor diffusivity, and *f*_T_. Water diffusivity inside restriction is assumed to be 1 × 10^−9^ mm^2^/s (*29*), but no tortuosity assumption is made. The convergence of two different sphere radii is ensured by different initializations (4 and 8 μm, respectively) and nonoverlapping variability range. The fitting algorithm is based on the lsqnonlin routine available in MATLAB. Average processing time per voxel is 5.9 s. The MCM is illustrated in fig. S2.

The low *b* value shell was used to fit the conventional tensor model and produce maps of the mean diffusivity. T_1_- and T_2_-weighted maps were also used to calculate the T_1_/T_2_ ratio, which is considered a proxy for myelination (*48*). T_2_* maps were calculated by fitting an exponential decay to the T_2_*-weighted images acquired at different echo times. In addition, for illustration purposes, the high-resolution anatomical scans were nonlinearly registered to a rat brain template (*49*) using an advanced normalization approach (*50*). Repeated-measures analysis of variance (ANOVA) was used to check for significant effect of the injection and of the group. Following significant effect, post hoc *t* tests were used to compare injected versus control hemisphere and corrected for multiple comparisons.

Similarly, human MRI data were preprocessed as follows. Motion, eddy current, and EPI distortions were corrected using FSL TOPUP and EDDY tools (*51*). Correction for gradient nonlinearities (*52*), signal drift (*53*), and Gibbs ringing artifacts (*54*) was also performed. All diffusion data were then registered to a skull-stripped (*55*) structural T_1_-weighted image using EPIREG (*51*). B0 scans were nonlinearly registered to a high-resolution human brain template (*56*) using an advanced normalization approach (*50*); then, the inverse transformation was applied to bring the Desikan GM parcellation in the single subject space. All masks were eroded by one voxel using the FSL command fslmaths to mitigate possible contamination from adjacent white matter. In addition, for each subject, the B0 scan was used for brain parcellation into gray and white matter, and only voxels with minimal white matter contamination (<5%) were retained for the analysis. Both intra- and intersubject coefficients of variations were calculated for each MRI measure. The Desikan parcellization was used to calculate mean and SD of the stick fraction and dispersion parameter in eight ROIs (hippocampus, cerebellum, substantia nigra, basal ganglia, thalamus, motor, frontal, and occipital cortices), which were correlated with postmortem histological staining for microglia, as reported in (*26*).

### Tissue processing and immunohistochemistry

Rats were deeply anesthetized with a lethal dose of sodium pentobarbital, 46 mg/kg, injected intraperitoneally (Dolethal, E.V.S.A. laboratories, Madrid, Spain). Rats were then perfused intracardially with 100 ml of 0.9% PBS and 100 ml of ice-cold 4% paraformaldehyde (PFA; BDH, Prolabo, VWR International, Lovaina, Belgium). Brains were immediately extracted from the skull and fixed for 1 hour in 4% PFA. Afterward, brains were included in 3% agarose/PBS (Sigma-Aldrich, Madrid, Spain) and cut in vibratome (VT 1000S, Leica, Wetzlar, Germany) in 50-μm-thick serial coronal sections.

Coronal sections were rinsed and permeabilized three times in 1× PBS with Triton X-100 at 0.5% (Sigma-Aldrich, Madrid, Spain) for 10 min each. Then, they were blocked in the same solution with 4% bovine serum albumin (Sigma-Aldrich, Madrid, Spain) and 2% goat serum donor herd (Sigma-Aldrich, Madrid, Spain) for 2 hours at room temperature. The slices were then incubated for one night at 4°C with primary antibodies for Iba-1 (1:1000; Wako Chemicals, Osaka, Japan), GFAP (1:1000; Sigma-Aldrich, Madrid, Spain), myelin basic protein (1:250; Merck Millipore, Massachusetts, USA), neurofilament 360Kd medium (1:250; Abcam, Cambridge, United Kingdom), and NeuN (1:250; Sigma-Aldrich, Madrid, Spain) to label microglia, astrocytes, myelin, neuron processes, and nuclei, respectively. The sections were subsequently incubated in specific secondary antibodies conjugated to the fluorescent probes, each at 1:500 (Thermo Fisher Scientific, Waltham, USA) for 2 hours at room temperature. Sections were then treated with 4′,6-Diamidine-2′-phenylindole dihydrochloride (DAPI) at 15 mM (Sigma-Aldrich, Madrid, Spain) during 15 min at room temperature. Last, sections were mounted on slides and covered with an anti-fading medium using a mix solution of 1:10 propyl-gallate:Mowiol (P3130, Sigma-Aldrich, Madrid, Spain; 475904, Merck Millipore, Massachusetts, USA). For myelin labeling, antigen retrieval was performed in 1% citrate buffer (Sigma-Aldrich, Madrid, Spain) and 0.05% Tween 20 (Sigma-Aldrich, Madrid, Spain), warmed to 80°C for protein unmasking. The rest of the steps were done as described above.

#### Imaging and data extraction

The tissue sections were then examined using a computer-assisted morphometry system consisting of a Leica DM4000 fluoresce microscope equipped with a QICAM Qimaging camera 22577 (Biocompare, San Francisco, USA) and Neurolucida morphometric software (MBF, Biosciences, VT, USA). Microglia were visualized and reconstructed under Leica HC PLC APO objective 20×/0.5, and astrocytes were visualized under Leica HC PLC APO objective 40×/0.75. Five cells per hippocampus per hemisphere were randomly selected for a total of 820 cells included for analysis (410 microglia, 410 astrocytes). Only cells that displayed intact and clear processes were included. Areas close to the injection scar were avoided. Cells were traced through the entire thickness of the sections, and trace information was then saved as three-dimensional (3D) reconstructions or rendered into a 2D diagram of each cell following analysis requirement.

Metric analysis of reconstructed cells was extracted using Neurolucida Explorer software (MBF, Biosciences, VT, USA) and Imaris (Bitplane, Belfast, United Kingdom): cell body perimeter, number of primary processes, number of nodes (branch points), complexity ([Sum of the terminal orders + Number of terminals] * [Total dendritic length/Number of primary dendrites]), fiber density and dendograms, cell size, and polar plots (fig. S5). Polar plots were analyzed to extract fiber orientation and the dispersion parameter in a plane parallel to the microscope, where higher values mean more uniform distribution of the fibers around the cell body. 3D convex analysis was performed to estimate astrocyte volume and overcome the limitations of GFAP labeling (*57*–*59*). The volume estimation in the analysis is defined as the area of the polygon created from straight lines connecting the most distal points of the astrocyte processes.

Density analysis was performed on 12-bit gray scale pictures acquired with the described system. ROIs were manually delineated following the Franklin and Paxinos rat brain atlas (*49*), covering the complete hippocampus in each hemisphere, for at least five slices per rat. Analysis was performed using Icy software (*59*) in a semiautomatic manner. The threshold for detection of positive nuclei was set for each condition, setting average nuclei size and a signal/noise ratio higher than 23%, according to Rayleigh criterion for resolution and discrimination between two points.

Myelin, neurofilament, and neural nuclei fluorescent analysis was also performed on pictures acquired with the described system and analyzed using Icy software (*59*). Two ROIs of 200 μm^2^ were placed per hippocampus per hemisphere in at least five slices per rat to obtain the corresponding intensity values.

#### Data analysis and statistics

The statistical analysis was done using GraphPad Prism 7 software (GraphPad Software Inc., La Jolla, CA, USA) and RStudio (RStudio 2015 Inc., Boston, MA). The presence of outlier values and parametric distribution were checked. We applied paired *t* tests for comparing, for each time point, control hemisphere versus injected hemisphere. Pearson’s correlation was used for regression analysis, and coefficients were transformed to apply Fisher’s 1925 test (*60*) for significant values. Polar analysis and dispersion estimation were performed to obtain the dispersion parameter (*61*).

## Supplementary Materials

### This PDF file includes:

Figs. S1 to S10 References

## Notes

View/request a protocol for this paper from *Bio-protocol*.
