# Supplementary material for: Mapping microglia and astrocyte activation in vivo using diffusion MRI
Source: Sci Adv. 2022 May 27;8(21):eabq2923. doi: 10.1126/sciadv.abq2923 (PMC9140964; doi:10.1126/sciadv.abq2923)
Supplement: Supplementary file 1 — Figs. S1 to S10 References [file sciadv.abq2923_sm.pdf]

Science Advances
AAAS

Supplementary Materials for

Mapping microglia and astrocyte activation in vivo using diffusion MRI

Raquel Garcia-Hernandez *et al.*

Corresponding author: Silvia De Santis, dsilvia@umh.es; Santiago Canals, scanals@umh.es

Sci. Adv. **8**, eabq2923 (2022)
DOI: 10.1126/sciadv.abq2923

This PDF file includes:

Figs. S1 to S10
References

Fig. S1: Experimental design and hypothesis based on previous literature

-

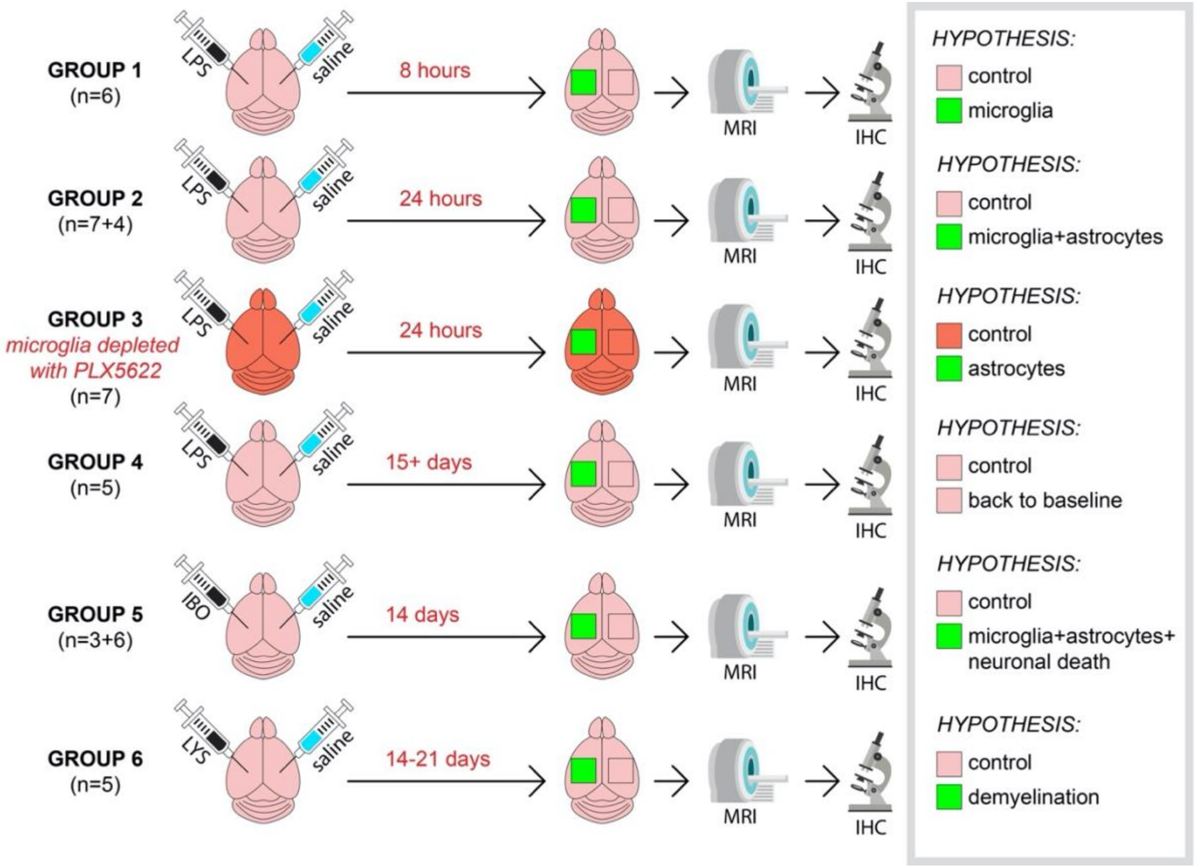

Experimental scheme showing bilateral stereotaxic injection of LPS (left hemisphere) / saline (right hemisphere) and the composition of the six groups: 6 animals were scanned 8 hours post-injection, 11 animals were scanned 24 hours post-injection, 8 animals were treated with PLX5622 for 7 days before the injection and then scanned 24 hours post-injection, and 5 animals were scanned 15 days or more post-injection. The last two rows show stereotaxic injection of ibotenic acid / lysolecithin (left hemisphere) or saline (right hemisphere) in a group of animals. Some (n=6) of the animals injected with ibotenic acid were previously treated with minocycline.

Fig. S2: Multi-compartment model

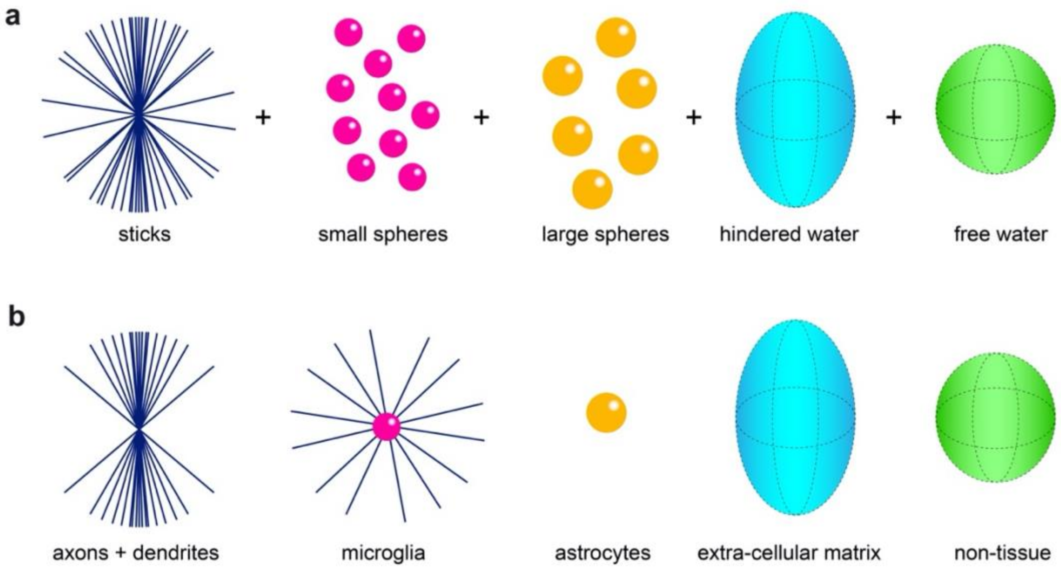

a, Multi-compartment tissue model comprising one compartment of water undergoing restricted diffusion in cylindric geometry (representing water trapped into cell ramifications) with a main orientation and a Watson dispersion term, two spherically restricted compartments, one extracellular space matrix, aligned with the main cylinder orientation and modelled as a tensor, and one compartment of water undergoing free diffusion. **b**, The compartments defined in **a** are combined to visually represent the different cell types constituting the parenchyma in our model: microglia, astrocytes, neurons, extracellular space.

Fig. S3 : Histology of myelin basic protein and neurofilament

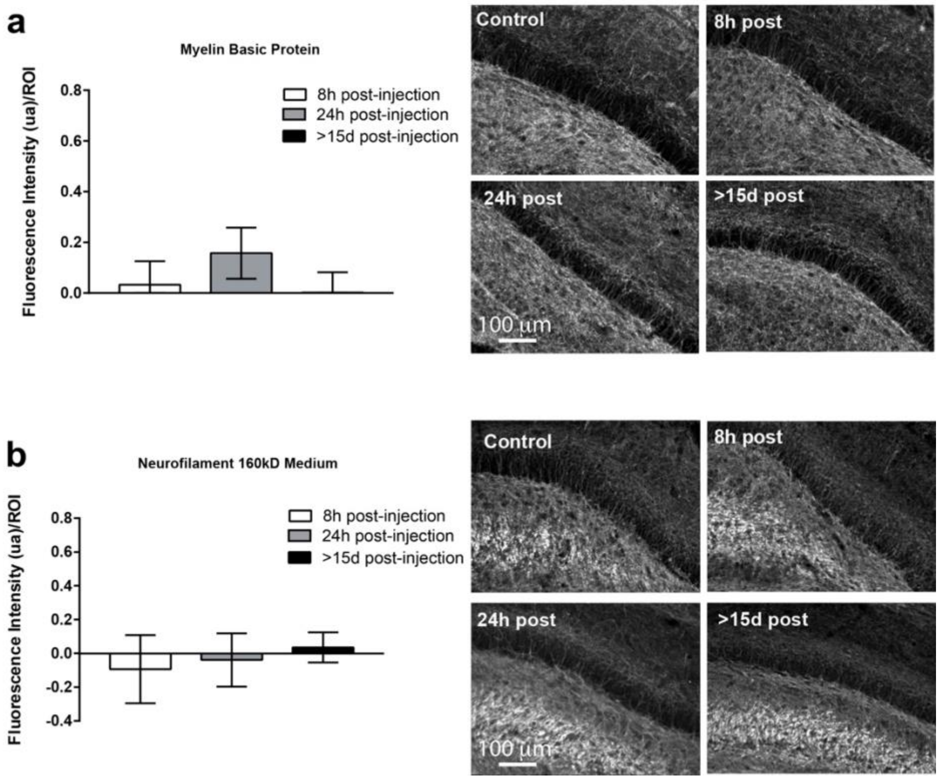

a, Myelin basic protein quantification results, plotted as mean and standard deviation, accompanied by representative microphotographs of the histological labelling at the different time points. **b**, same for Neurofilament. Scale bar=100 µm.

Fig. S4: conventional MRI parameters in LPS-injected hemisphere versus control

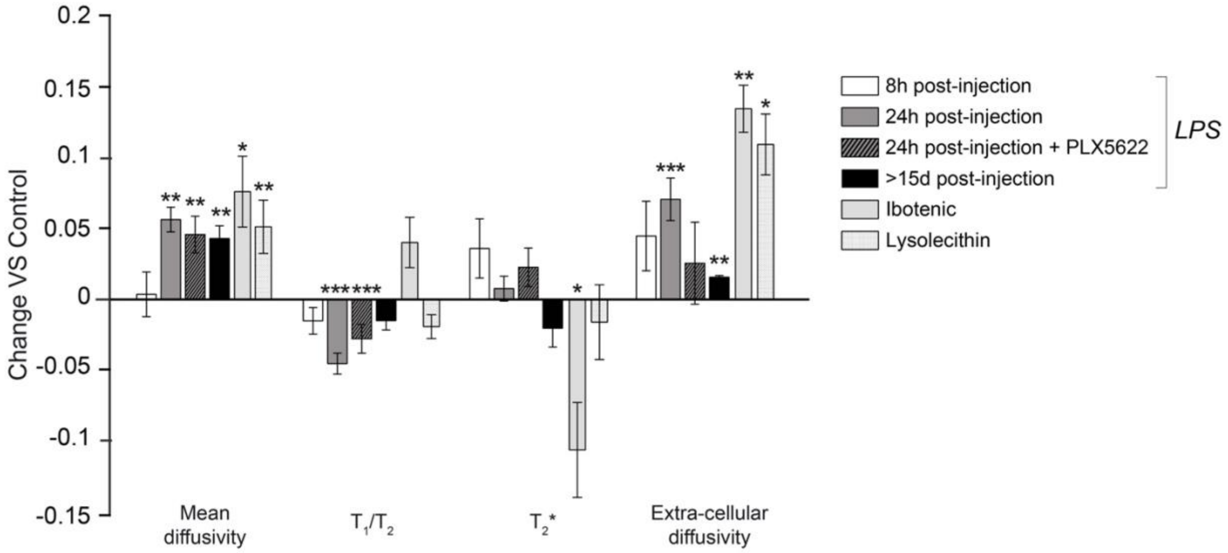

Normalized change $(P_{injected}-P_{control})/P_{control}$ between MRI-derived mean diffusivity, T_1/T_2, T_2* and extracellular diffusivity from the multi-compartment model, calculated in the injected vs control hemisphere for the astrocyte compartment (shown in the insert). Asterisks represent significant paired difference between injected and control. Error bars represent standard deviation.

Fig. S5: Histology main methods and morphometric features obtained

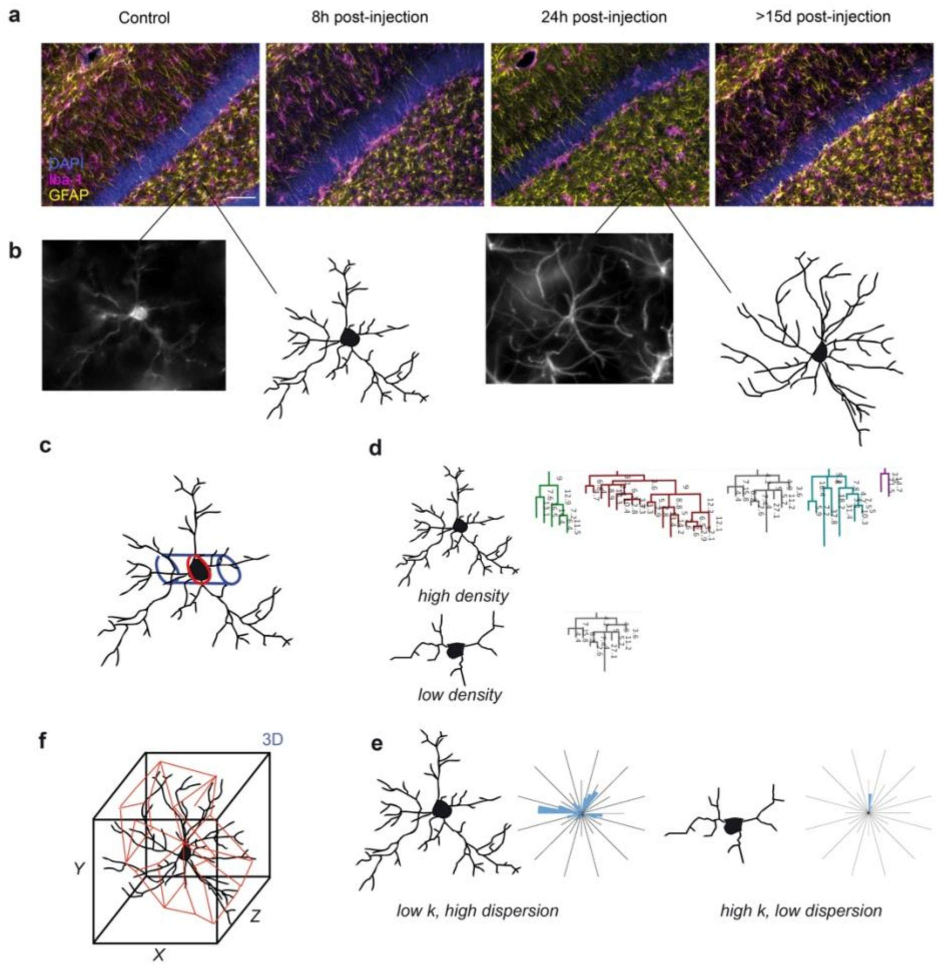

a, Representative microphotographs of the histological labelling at the different time points. Green= Iba1 (microglia), red= GFAP (astrocytes), Blue= DAPI (Cell nuclei). Scale bar= 100 µm **b**, zoom in of a microglia and astrocyte and their 3D reconstruction for morphometric analysis. **c,** microglia cell size extraction by cross-sectional area determination. **d,** Microglia's fiber density extraction, showing two representative cases of high and low densities. **f,** Astrocyte's convex hull extraction. **e,** Microglia's polar plots for fiber orientation analysis, showing two representative cases of low Watson dispersion parameter k (indicating high fiber dispersion along the main orientation) and high k (indicating low fiber dispersion).

Fig. S6: Microglia density at the different timepoints after LPS injections

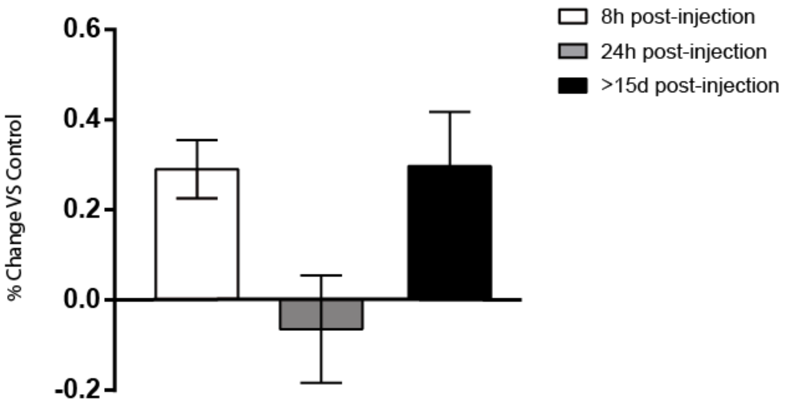

Normalized change ($P_{injected}$-$P_{control}$)/$P_{control}$ in microglial density at the different time points, calculated in the LPS-injected vs control hemisphere for the whole hippocampus. Error bars represent standard deviation.

Fig. S7: Microglia depletion in PLX5622-treated versus control animals

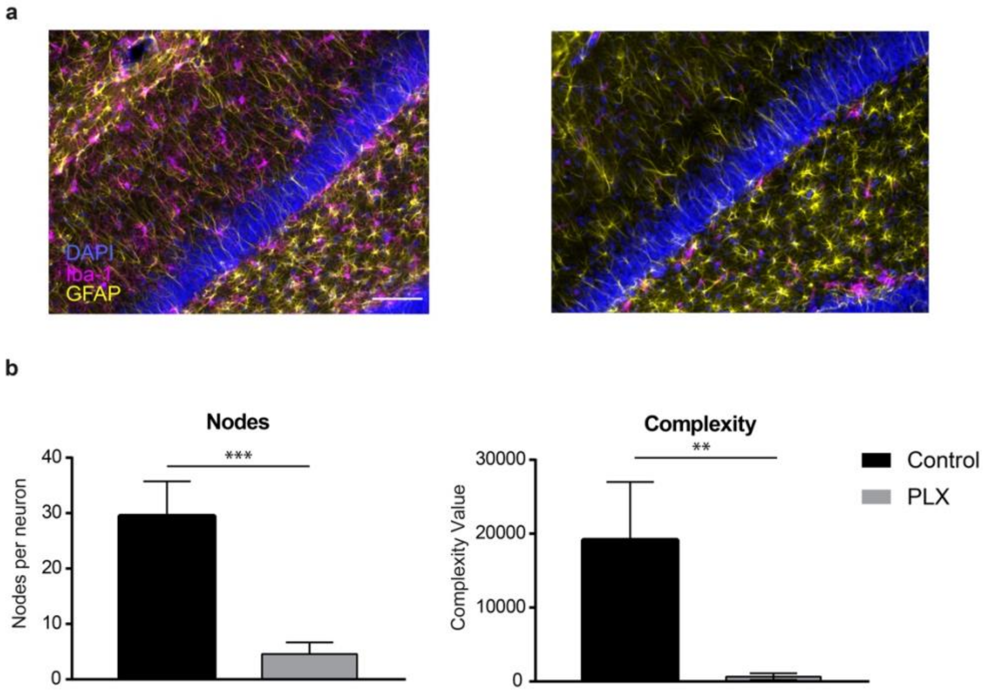

a, Representative microphotographs of a control rat and PLX5622-treated rat, respectively. Green= Iba1 (microglia), red= GFAP (astrocytes), Blue= DAPI (Cell nuclei). Scale bar= 100 μm **b**, Nodes number and complexity quantifications of microglia in both cases, plotted as mean and standard deviation. Asterisks represent significant t-test difference between injected and control.

Fig. S8: Whole GM average maps of stick fraction in rats

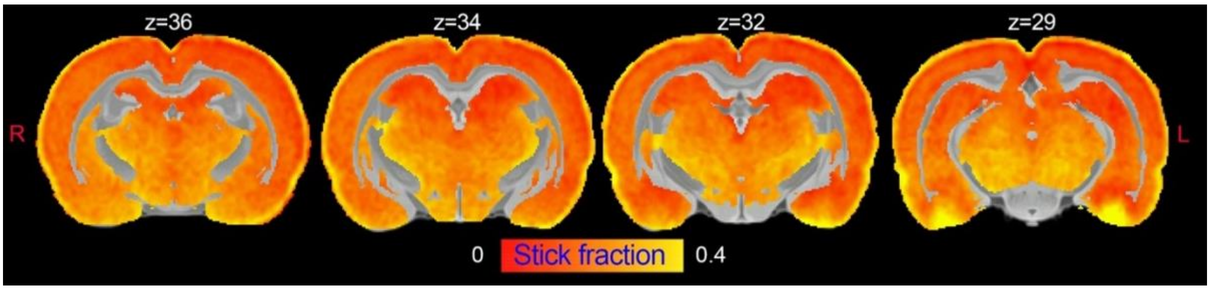

Stick fraction according to the multi-compartment model normalized to the rat brain template defined in (*49*), masked for grey matter tissue, and averaged across subjects.

Fig. S9: GFAP staining in rats injected with ibotenic acid

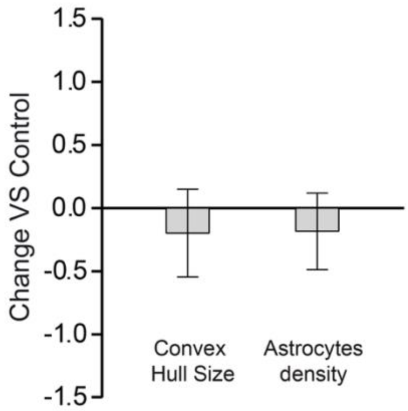

Normalized change ($(P_{injected}-P_{control})/P_{control}$) in GFAP convex hull size and density, calculated in the ibotenic-injected vs control hemisphere for the whole hippocampus. Error bars represent standard deviation.

Fig. S10: Dispersion parameter k in condition of microglia activation, neuronal loss and demyelination

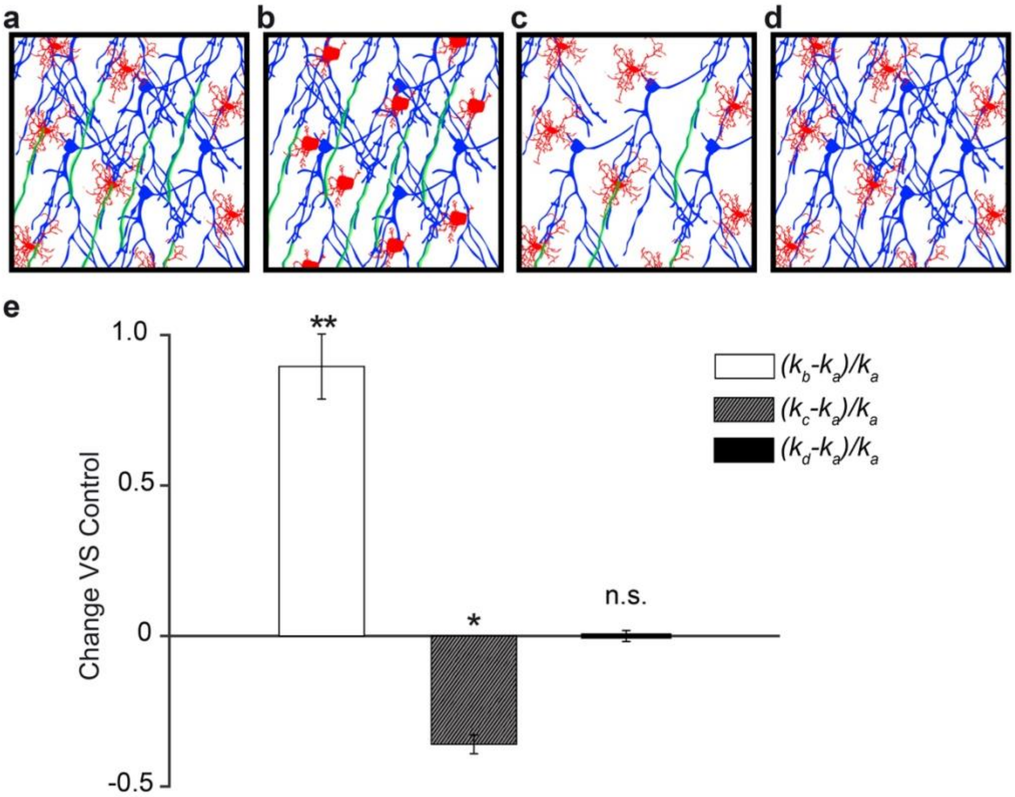

MRI synthetic signal was generated for a geometry composed by microglia, astrocytes and neurons. Microglia and dendrites were characterized by size and processes/dendrite dispersions as measured in (*62*) (microglia) and (*63*)(neurons), as schematized in panel **a**. A myelin compartment was also included, with a g-ratio of 0.7. MRI synthetic signal was generated in a similar substrate, but with a 50% reduction of microglia ramification, and a 10% increase in cell body size as schematized in panel **b**. A third substrate, instead, simulated a 50% reduction of dendrites, as illustrated in panel **c**. Finally, a fourth substrate was generated with no myelin and increased extra-axonal diffusivity. 2*10^3 noisy repetitions (Rician noise) were generated, and the resulting signal was fitted using the MCM to extract the dispersion parameter k. Normalized difference in k ((k_x-k_a)/k_a, were x=b,c,d) are reported in panel **e**. Asterisks represent significant differences in the 1-sample t-test (*=P<0.05, **=P<0.01).

REFERENCES AND NOTES

1. R. M. Ransohoff, How neuroinflammation contributes to neurodegeneration. *Science* **353**, 777–783 (2016).

2. H. Lassmann, J. van Horssen, D. Mahad, Progressive multiple sclerosis: Pathology and pathogenesis. *Nat. Rev. Neurol.* **8**, 647–656 (2012).

3. C. K. Glass, K. Saijo, B. Winner, M. C. Marchetto, F. H. Gage, Mechanisms underlying inflammation in neurodegeneration. *Cell* **140**, 918–934 (2010).

4. C. Janus, J. Pearson, J. McLaurin, P. M. Mathews, Y. Jiang, S. D. Schmidt, M. A. Chishti, P. Horne, D. Heslin, J. French, H. T. J. Mount, R. A. Nixon, M. Mercken, C. Bergeron, P. E. Fraser, P. St George-Hyslop, D. Westaway, Aβ peptide immunization reduces behavioural impairment and plaques in a model of Alzheimer's disease. *Nature* **408**, 979–982 (2000).

5. N. Yahfoufi, C. Matar, N. Ismail, Adolescence and aging: Impact of adolescence inflammatory stress and microbiota alterations on brain development, aging, and neurodegeneration. *J. Gerontol. Ser. A.* **75**, 1251–1257 (2020).

6. B. Wilbanks, L. Maher, M. Rodriguez, Glial cells as therapeutic targets in progressive multiple sclerosis. *Expert Rev. Neurother.* **19**, 481–494 (2019).

7. M. Zella, J. Metzdorf, F. Ostendorf, F. Maass, S. Muhlack, R. Gold, A. Haghikia, L. Tönges, Novel Immunotherapeutic approaches to target alpha-synuclein and related neuroinflammation in Parkinson's disease. *Cell* **8**, 105 (2019).

8. S. Amor, F. Puentes, D. Baker, P. van der Valk, Inflammation in neurodegenerative diseases. *Immunology* **129**, 154–169 (2010).

9. D. R. J. Owen, R. N. Gunn, E. A. Rabiner, I. Bennacef, M. Fujita, W. C. Kreisl, R. B. Innis, V. W. Pike, R. Reynolds, P. M. Matthews, C. A. Parker, Mixed-affinity binding in humans with 18-kDa translocator Protein ligands. *J. Nucl. Med.* **52**, 24–32 (2011).

10. T. R. Guilarte, TSPO in diverse CNS pathologies and psychiatric disease: A critical review and a way forward. *Pharmacol. Ther.* **194**, 44–58 (2019).

11. P. J. Basser, C. Pierpaoli, Microstructural and physiological features of tissues elucidated by quantitative-diffusion-tensor MRI. *J. Magn. Reson. Ser. B* **111**, 209–219 (1996).

12. S. Y. Yi, B. R. Barnett, M. Torres-Velázquez, Y. Zhang, S. A. Hurley, P. A. Rowley, D. Hernando, J.-P. J. Yu, Detecting microglial density with quantitative multi-compartment diffusion MRI. *Front. Neurosci.* **13**, 81 (2019).

13. H. N. Noristani, H. Boukhaddaoui, G. Saint-Martin, P. Auzer, R. Sidiboulenouar, N. Lonjon, E. Alibert, N. Tricaud, C. Goze-Bac, C. Coillot, F. E. Perrin, A combination of ex vivo diffusion MRI and multiphoton to study microglia/monocytes alterations after spinal cord injury. *Front. Aging Neurosci.* **9**, 230 (2017).

14. M. Taquet, A. Jankovski, G. Rensonnet, D. Jacobs, A. des Rieux, B. Macq, S. K. Warfield, B. Scherrer, Extra-axonal restricted diffusion as an in-vivo marker of reactive microglia. *Sci. Rep.* **9**, 13874 (2019).

15. D. Barazany, P. J. Basser, Y. Assaf, In vivo measurement of axon diameter distribution in the corpus callosum of rat brain. *Brain* **132**, 1210–1220 (2009).

16. A. M. Espinosa-Oliva, R. M. de Pablos, A. J. Herrera, in *Microglia*, B. Joseph, J. L. Venero, Eds. (Humana Press, 2013), vol. 1041 of *Methods in Molecular Biology*, pp. 295–305.

17. H.-K. Jeong, I. Jou, E. Joe, Systemic LPS administration induces brain inflammation but not dopaminergic neuronal death in the substantia nigra. *Exp. Mol. Med.* **42**, 823 (2010).

18. J. Han, R. A. Harris, X.-M. Zhang, An updated assessment of microglia depletion: Current concepts and future directions. *Mol. Brain* **10**, 25 (2017).

19. J. Drouin-Ouellet, A.-L. Brownell, M. Saint-Pierre, C. Fasano, V. Emond, L.-E. Trudeau, D. Lévesque, F. Cicchetti, Neuroinflammation is associated with changes in glial mGluR5 expression and the development of neonatal excitotoxic lesions. *Glia* **59**, 188–199 (2011).

20. R. H. Woodruff, R. J. M. Franklin, Demyelination and remyelination of the caudal cerebellar peduncle of adult rats following stereotaxic injections of lysolecithin, ethidium bromide, and complement/anti-galactocerebroside: A comparative study. *Glia* **25**, 216–228 (1999).

21. A. Verkhratsky, A. Butt, *Glial Neurobiology* (John Wiley & Sons Ltd., 2007).

22. J. Morrens, W. Van Den Broeck, G. Kempermann, Glial cells in adult neurogenesis. *Glia* **60**, 159–174 (2012).

23. O. Pasternak, N. Sochen, Y. Gur, N. Intrator, Y. Assaf, Free water elimination and mapping from diffusion MRI. *Magn. Reson. Med.* **62**, 717–730 (2009).

24. S. Cheng, J. Hou, C. Zhang, C. Xu, L. Wang, X. Zou, H. Yu, Y. Shi, Z. Yin, G. Chen, Minocycline reduces neuroinflammation but does not ameliorate neuron loss in a mouse model of neurodegeneration. *Sci. Rep.* **5**, 10535 (2015).

25. D. K. Jones, D. C. Alexander, R. Bowtell, M. Cercignani, F. Dell'Acqua, D. J. McHugh, K. L. Miller, M. Palombo, G. J. M. Parker, U. S. Rudrapatna, C. M. W. Tax, Microstructural imaging of the human brain with a 'super-scanner': 10 key advantages of ultra-strong gradients for diffusion MRI. *Neuroimage* **182**, 8–38 (2018).

26. M. Mittelbronn, K. Dietz, H. J. Schluesener, R. Meyermann, Local distribution of microglia in the normal adult human central nervous system differs by up to one order of magnitude. *Acta Neuropathol.* **101**, 249–255 (2001).

27. S. De Santis, S. Canals, Non-invasive MRI windows to neuroinflammation. *Neuroscience* **403**, 1–3 (2019).

28. S. N. Jespersen, C. D. Kroenke, L. Østergaard, J. J. H. Ackerman, D. A. Yablonskiy, Modeling dendrite density from magnetic resonance diffusion measurements. *Neuroimage* **34**, 1473–1486 (2007).

29. H. Zhang, T. Schneider, C. A. Wheeler-Kingshott, D. C. Alexander, NODDI: Practical in vivo neurite orientation dispersion and density imaging of the human brain. *Neuroimage* **61**, 1000–1016 (2012).

30. M. Palombo, A. Ianus, M. Guerreri, D. Nunes, D. C. Alexander, N. Shemesh, H. Zhang, SANDI: A compartment-based model for non-invasive apparent soma and neurite imaging by diffusion MRI. *Neuroimage* **215**, 116835 (2020).

31. C. Ligneul, M. Palombo, E. Hernández-Garzón, M.-A. Carrillo-de Sauvage, J. Flament, P. Hantraye, E. Brouillet, G. Bonvento, C. Escartin, J. Valette, Diffusion-weighted magnetic resonance spectroscopy enables cell-specific monitoring of astrocyte reactivity in vivo. *Neuroimage* **191**, 457–469 (2019).

32. D. Gomez-Nicola, N. L. Fransen, S. Suzzi, V. H. Perry, Regulation of microglial proliferation during chronic neurodegeneration. *J. Neurosci.* **33**, 2481–2493 (2013).

33. S. De Santis, T. Granberg, R. Ouellette, C. A. Treaba, E. Herranz, Q. Fan, C. Mainero, N. Toschi, Evidence of early microstructural white matter abnormalities in multiple sclerosis from multi-shell diffusion MRI. *NeuroImage Clin.* **22**, 101699 (2019).

34. S. Collorone, N. Cawley, F. Grussu, F. Prados, F. Tona, A. Calvi, B. Kanber, T. Schneider, L. Kipp, H. Zhang, D. C. Alexander, A. J. Thompson, A. Toosy, C. A. G. Wheeler-Kingshott, O. Ciccarelli, Reduced neurite density in the brain and cervical spinal cord in relapsing–remitting multiple sclerosis: A NODDI study. *Mult. Scler.* **26**, 1647–1657 (2020).

35. N. Stikov, J. S. W. Campbell, T. Stroh, M. Lavelée, S. Frey, J. Novek, S. Nuara, M.-K. Ho, B. J. Bedell, R. F. Dougherty, I. R. Leppert, M. Boudreau, S. Narayanan, T. Duval, J. Cohen-Adad, P.-A. Picard, A. Gasecka, D. Côté, G. B. Pike, In vivo histology of the myelin g-ratio with magnetic resonance imaging. *Neuroimage* **118**, 397–405 (2015).

36. A. Horowitz, D. Barazany, I. Tavor, G. Yovel, Y. Assaf, Response to the comments on the paper by Horowitz et al. (2014). *Brain Struct. Funct.* **220**, 1791–1792 (2015).

37. G. Flügge, C. Araya-Callis, E. Garea-Rodriguez, C. Stadelmann-Nessler, E. Fuchs, NDRG2 as a marker protein for brain astrocytes. *Cell Tissue Res.* **357**, 31–41 (2014).

38. Z. Zhang, Z. Ma, W. Zou, H. Guo, M. Liu, Y. Ma, L. Zhang, The appropriate marker for astrocytes: Comparing the distribution and expression of three astrocytic markers in different mouse cerebral regions. *Biomed. Res. Int.* **2019**, 1–15 (2019).

39. R. M. Ransohoff, A polarizing question: Do M1 and M2 microglia exist? *Nat. Neurosci.* **19**, 987–991 (2016).

40. T. V. Veenith, E. Carter, J. Grossac, V. F. J. Newcombe, J. G. Outtrim, V. Lupson, G. B. Williams, D. K. Menon, J. P. Coles, Inter subject variability and reproducibility of diffusion tensor imaging within and between different imaging sessions. *PLOS ONE* **8**, e65941 (2013).

41. S. De Santis, M. Drakesmith, S. Bells, Y. Assaf, D. K. Jones, Why diffusion tensor MRI does well only some of the time: Variance and covariance of white matter tissue microstructure attributes in the living human brain. *Neuroimage* **89**, 35–44 (2014).

42. H.-H. Lee, A. Papaioannou, D. S. Novikov, E. Fieremans, In vivo observation and biophysical interpretation of time-dependent diffusion in human cortical gray matter. *Neuroimage* **222**, 117054 (2020).

43. M. Nilsson, J. Lätt, D. van Westen, S. Brockstedt, S. Lasič, F. Ståhlberg, D. Topgaard, Noninvasive mapping of water diffusional exchange in the human brain using filter-exchange Imaging. *Magn. Reson. Med.* **69**, 1572–1580 (2013).

44. M. Afzali, M. Nilsson, M. Palombo, D. K. Jones, SPHERIOUSLY? The challenges of estimating sphere radius non-invasively in the human brain from diffusion MRI. *Neuroimage* **237**, 118183 (2021).

45. S. De Santis, A. Cosa-Linan, R. Garcia-Hernandez, L. Dmytrenko, L. Vargova, I. Vorisek, S. Stopponi, P. Bach, P. Kirsch, F. Kiefer, R. Ciccocioppo, E. Sykova, D. Moratal, W. H. Sommer, S. Canals, Chronic alcohol consumption alters extracellular space geometry and transmitter diffusion in the brain. *Sci. Adv.* **6**, eaba0154 (2020).

46. C. H. Neuman, Spin echo of spins diffusing in a bounded medium. *J. Chem. Phys.* **60**, 4508–4511 (1974).

47. M. B. Hansen, S. N. Jespersen, L. A. Leigland, C. D. Kroenke, Using diffusion anisotropy to characterize neuronal morphology in gray matter: The orientation distribution of axons and dendrites in the NeuroMorpho.org database. *Front. Integr. Neurosci.* **7**, 31 (2013).

48. M. F. Glasser, D. C. Van Essen, Mapping human cortical areas in vivo based on myelin content as revealed by T1- and T2-weighted MRI. *J. Neurosci.* **31**, 11597–11616 (2011).

49. J. Buttner-Ennever, The rat brain in stereotaxic coordinates, 3rd edn. By George Paxinos and Charles Watson. (Pp. xxxiii+80; illustrated; f$69.95 paperback; ISBN 0 12 547623; comes with CD-ROM.) San Diego: Academic Press. 1996. *J. Anat.* **191**, 315–317 (1997).

50. A. Klein, J. Andersson, B. A. Ardekani, J. Ashburner, B. Avants, M.-C. Chiang, G. E. Christensen, D. L. Collins, J. Gee, P. Hellier, J. H. Song, M. Jenkinson, C. Lepage, D. Rueckert, P. Thompson, T. Vercauteren, R. P. Woods, J. J. Mann, R. V. Parsey, Evaluation of 14 nonlinear deformation algorithms applied to human brain MRI registration. *Neuroimage* **46**, 786–802 (2009).

51. J. L. R. Andersson, S. N. Sotiropoulos, An integrated approach to correction for off-resonance effects and subject movement in diffusion MR imaging. *Neuroimage* **125**, 1063–1078 (2016).

52. M. F. Glasser, S. N. Sotiropoulos, J. A. Wilson, T. S. Coalson, B. Fischl, J. L. Andersson, J. Xu, S. Jbabdi, M. Webster, J. R. Polimeni, D. C. Van Essen, M. Jenkinson, The minimal preprocessing pipelines for the human connectome project. *Neuroimage* **80**, 105–124 (2013).

53. S. B. Vos, C. M. W. Tax, P. R. Luijten, S. Ourselin, A. Leemans, M. Froeling, The importance of correcting for signal drift in diffusion MRI. *Magn. Reson. Med.* **77**, 285–299 (2017).

54. E. Kellner, B. Dhital, V. G. Kiselev, M. Reisert, Gibbs-ringing artifact removal based on local subvoxel-shifts. *Magn. Reson. Med.* **76**, 1574–1581 (2016).

55. S. M. Smith, Fast robust automated brain extraction. *Hum. Brain Mapp.* **17**, 143–155 (2002).

56. R. S. Desikan, F. Ségonne, B. Fischl, B. T. Quinn, B. C. Dickerson, D. Blacker, R. L. Buckner, A. M. Dale, R. P. Maguire, B. T. Hyman, M. S. Albert, R. J. Killiany, An automated labeling system for subdividing the human cerebral cortex on MRI scans into gyral based regions of interest. *Neuroimage* **31**, 968–980 (2006).

57. J. Haseleu, E. Anlauf, S. Blaess, E. Endl, A. Derouiche, Studying subcellular detail in fixed astrocytes: Dissociation of morphologically intact glial cells (DIMIGs). *Front. Cell. Neurosci.* **7**, 54 (2013).

58. P. M. Kulkarni, E. Barton, M. Savelonas, R. Padmanabhan, Y. Lu, K. Trett, W. Shain, J. L. Leasure, B. Roysam, Quantitative 3-D analysis of GFAP labeled astrocytes from fluorescence confocal images. *J. Neurosci. Methods* **246**, 38–51 (2015).

59. F. de Chaumont, S. Dallongeville, N. Chenouard, N. Hervé, S. Pop, T. Provoost, V. Meas-Yedid, P. Pankajakshan, T. Lecomte, Y. Le Montagner, T. Lagache, A. Dufour, J.-C. Olivo-Marin, Icy: An open bioimage informatics platform for extended reproducible research. *Nat. Methods* **9**, 690–696 (2012).

60. R. A. Fisher, in *Breakthroughs in Statistics*, S. Kotz, N. L. Johnson, Eds. (Springer New York, 1992), pp. 66–70.

61. J. Mollink, M. Kleinnijenhuis, A.-M. van Cappellen van Walsum, S. N. Sotiropoulos, M. Cottaar, C. Mirfin, M. P. Heinrich, M. Jenkinson, M. Pallebage-Gamarallage, O. Ansorge, S. Jbabdi, K. L. Miller, Evaluating fibre orientation dispersion in white matter: Comparison of diffusion MRI, histology and polarized light imaging. *Neuroimage* **157**, 561–574 (2017).

62. I. E. Papageorgiou, A. F. Fetani, A. Lewen, U. Heinemann, O. Kann, Widespread activation of microglial cells in the hippocampus of chronic epileptic rats correlates only partially with neurodegeneration. *Brain Struct. Funct.* **220**, 2423–2439 (2015).

63. G. M. Arisi, N. Garcia-Cairasco, Doublecortin-positive newly born granule cells of hippocampus have abnormal apical dendritic morphology in the pilocarpine model of temporal lobe epilepsy. *Brain Res.* **1165**, 126–134 (2007).
